# The Potential Role of Small-Molecule PERK Inhibitor LDN-0060609 in Primary Open-Angle Glaucoma Treatment

**DOI:** 10.3390/ijms22094494

**Published:** 2021-04-26

**Authors:** Wioletta Rozpędek-Kamińska, Grzegorz Galita, Natalia Siwecka, Steven L. Carroll, John Alan Diehl, Ewa Kucharska, Dariusz Pytel, Ireneusz Majsterek

**Affiliations:** 1 Department of Clinical Chemistry and Biochemistry, Medical University of Lodz, 90-419 Lodz, Poland; wioletta.rozpedek@umed.lodz.pl (W.R.-K.); grzegorz.galita@umed.lodz.pl (G.G.); natalia.siwecka@stud.umed.lodz.pl (N.S.); 2 Department of Pathology and Laboratory Medicine, Medical University of South Carolina, Charleston, SC 29425, USA; carrolst@musc.edu; 3 Hollings Cancer Center, Department of Biochemistry and Molecular Biology, Medical University of South Carolina, Charleston, SC 29425, USA; jad283@case.edu; 4 Department of Biochemistry, School of Medicine, Case Western Reserve University, Cleveland, OH 44106, USA; 5 Department of Gerontology, Geriatrics and Social Work, Jesuit University Ignatianum, 31-501 Krakow, Poland; ewa.kucharska@ignatianum.edu.pl

**Keywords:** glaucoma, PERK, eIF2α, endoplasmic reticulum stress, unfolded protein response, apoptosis, PERK inhibitor, glaucoma treatment

## Abstract

Primary open-angle glaucoma (POAG) constitutes the most common type of glaucoma. Emerging evidence suggests that Endoplasmic Reticulum (ER) stress and the protein kinase RNA-like endoplasmic reticulum kinase (PERK)-mediated Unfolded Protein Response (UPR) signaling pathway play a key role in POAG pathogenesis. Thus, the main aim of the study was to evaluate the effectiveness of the PERK inhibitor LDN-0060609 in cellular model of glaucoma using primary human trabecular meshwork (HTM) cells. To evaluate the level of the ER stress marker proteins, Western blotting and TaqMan gene expression assay were used. The cytotoxicity was measured by XTT, LDH assays and Giemsa staining, whereas genotoxicity via comet assay. Changes in cell morphology were assessed by phase-contrast microscopy. Analysis of apoptosis was performed by caspase-3 assay and flow cytometry (FC), whereas cell cycle progression by FC. The results obtained have demonstrated that LDN-0060609 triggered a significant decrease of ER stress marker proteins within HTM cells with induced ER stress conditions. Moreover, LDN-0060609 effectively increased viability, reduced DNA damage, increased proliferation, restored normal morphology, reduced apoptosis and restored normal cell cycle distribution of HTM cells with induced ER stress conditions. Thereby, PERK inhibitors, such as LDN-0060609, may provide an innovative, ground-breaking treatment strategy against POAG.

## 1. Introduction

Glaucoma, that affects more than 70 million people worldwide, is commonly known as a chronic eye disease characterized by a progressive neurodegeneration of the optic nerve and rapid loss of retinal ganglion cells (RGCs), that directly lead to irreversible loss of vision [1]. Currently, despite many studies, an etiology of glaucoma still remains poorly understood as well as factors that may contribute to its progression have not been fully characterized [2,3]. Glaucoma, a ‘silent thief of sight’, may remain asymptomatic until later stages of the disease occur, and thereby there is a high frequency of undiagnosed glaucoma cases worldwide [4,5]. Glaucoma which is second only to cataracts as the cause of blindness globally is characterized by a high prevalence, accounting for 12.3% of blindness worldwide [6]. It is estimated that in 2040, the number of glaucoma-affected patients may reach up to 111.8 million worldwide [7]. There are several types of glaucoma, whereas primary glaucoma occurs in the absence of an identifiable secondary cause, such as pseudoexfoliation, pigment dispersion or chronic uveitis. Glaucoma, based on the anterior chamber angle morphology, is commonly classified into open-angle glaucoma (OAG) and angle-closure glaucoma (ACG) [8,9]. Both OAG and ACG may constitute a primary disease [3]. Primary open-angle glaucoma (POAG) is predominantly classified based on the age of onset, thereby it is divided into primary congenital glaucoma with onset up to 3 years of age, juvenile open-angle glaucoma (JOAG), affecting individuals between 10–35 years of age, as well as adult-onset POAG, affecting individuals over the age of 35. It has been reported that the latter is the most common form and it does not have a clear pattern of inheritance [10]. It has been demonstrated that there are numerous risk factors that may contribute to POAG development, including, among others, high intraocular pressure (IOP), positive family history, advanced age, black race, increased cup–disk ratio (CDR), CDR asymmetry, disc hemorrhage as well as corticosteroid use, whereas high IOP is considered a major factor in POAG development [11,12,13,14,15].

There are several lines of evidence that POAG pathogenesis may be strictly correlated at the molecular level with the Endoplasmic Reticulum (ER) stress conditions and subsequent activation of the protein kinase RNA-like endoplasmic reticulum kinase (PERK)-mediated Unfolded Protein Response (UPR) signaling pathway [16,17,18,19]. Worthy of notice, PERK pathway could be also modulate by carbon monoxide, which is implicated in POAG [20]. ER is a dynamic organelle, which is directly responsible for crucial functions for maintaining cellular homeostasis [21], including protein synthesis, transport and folding, lipid and steroid synthesis, carbohydrate metabolism as well as calcium storage [22]. ER stress-mediated induction of the UPR is directly correlated with activation of the three ER transmembrane sensor proteins such as PERK, inositol requiring enzyme 1 (IRE1) and activating transcription factor 6 (ATF6) [23,24,25]. ER homeostasis is maintained by ER chaperones such as glucose-regulated protein 78 (GRP78) [26,27], whereas it has been demonstrated that various factors may trigger an accumulation of the unfolded and misfolded proteins within the ER lumen, disrupting therefore ER homeostasis [28,29,30]. Under prolonged and severe ER stress conditions, PERK undergoes oligomerization and autophosphorylation, that in turn leads to the phosphorylation of the main PERK substrate, namely eukaryotic initiation factor 2α (eIF2α). Above-mentioned molecular events result in the attenuation of global protein translation as well as enhanced translation of only selective proteins such as activating transcription factor 4 (ATF4), that under mild or moderate ER stress conditions is responsible for restoration of the cellular homeostasis, whereas under severe and chronic ER stress conditions may activate CCAAT-enhancer-binding protein homologous protein (CHOP)-mediated apoptotic cell death [31,32,33,34]. It has been reported that CHOP-dependent cell death occurs upon markedly increased expression of pro-apoptotic proteins including B-cell lymphoma-2 (BCL-2), growth arrest and DNA damage-inducible protein (GADD34), endoplasmic reticulum oxidoreductin 1 (ERO1), or tribbles-related protein 3 (TRB3). CHOP overexpression may induce apoptosis via activation and mitochondrial translocation of Bax [35,36,37]. The pro-apoptotic Bax protein is regarded as a key mediator of RGCs apoptosis [38], and elevated levels of Bax were found in the axons of the optic nerve in absolute glaucoma patients [39].

Currently available glaucoma treatment is limited and generates various side effects in patients. The major treatment methods for POAG are primarily based only on reducing aqueous humor (AH) formation, enhancing uveoscleral or conventional outflow or lowering IOP. Thus, above-mentioned treatment strategies for POAG are symptomatic and may only slow a disease progression, whereas they cannot lead to the prevention of vision loss [2,3]; therefore, other treatment strategies are needed to reduce or reverse the progressive neurodegeneration in POAG patients. This is of particular importance especially in terms of treatment-resistant glaucoma, so as to avoid the surgical treatment, that is considered invasive, is not always effective and may be associated with irreversible side effects [40]. Based on the latest research data implying that ER stress and activation of the PERK-dependent UPR signaling pathway may be closely correlated with POAG pathogenesis at the molecular level, the main aim of the present study was to evaluate the effectiveness of the small-molecule PERK inhibitor LDN-0060609 in an in vitro cellular experimental model of glaucoma, since targeting of the components of the UPR signaling branches may contribute to the development of an innovative, ground-breaking treatment strategy against POAG. Previously, it has been demonstrated that PERK inhibitor LDN-0060609 (named as AMD), depending on pH, may exist as ketone, enol and enolate in aqueous solution. Both oxidative and reductive stress are directly correlated with the pathogenesis of multiple human diseases including POAG, being one of the neurodegenerative disorders. Interestingly, on the basis of radiolysis studies, it has been demonstrated that the triketone LDN-0060609 may play an important role in scavenging the excessive amount of free radicals. Thus, investigated herein small-molecule LDN-0060609 compound constitutes an effective free radical scavenger, which is an important aspect considering its potential use in the POAG treatment [41].

## 2. Results

### 2.1. Evaluation of the PERK Inhibitor LDN-0060609 Activity

**Figure 1:**
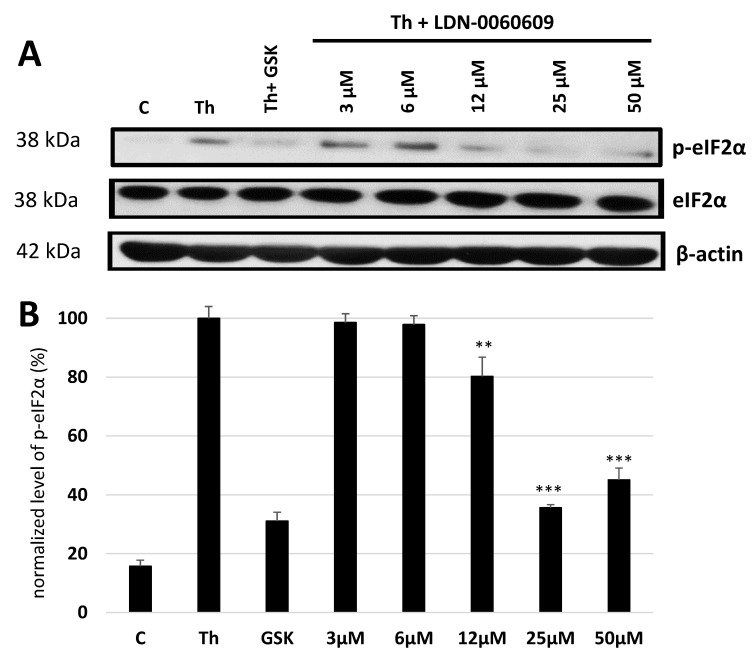
Analysis of the LDN-0060609 and GSK2606414 inhibitory compounds activity against eIF2α phosphorylation by the Western blot technique (**A**) and normalized level of p-eIF2α measured by optical densitometry (**B**). Each of the statistical analysis in individual experiments was based on the results of three independent tests. Data are expressed as mean ± SE (*n* = 3). On the graphs, the differences were statistically significant as follows: ** *p* < 0.01, *** *p* < 0.001 versus the positive control (Th). C—negative control, untreated HTM cells, Th—thapsigargin.

To assess the cellular response of the primary human trabecular meshwork (HTM) cells with activated ER stress conditions to treatment with the investigated PERK inhibitor LDN-0060609, the PERK enzyme activity was evaluated via measurement of the level of the phosphorylated form of eIF2α, as the main substrate of PERK, by the Western blot technique. The percentage of p-eIF2α was quantified by densitometry analysis. Treatment with thapsigargin (Th), an ER stress inducer, triggered an increased phosphorylation of the eIF2α compared to control cells cultured in a complete medium. Interestingly, obtained results demonstrated that PERK inhibitor LDN-0060609 significantly reduced ER stress-dependent phosphorylation of the eIF2α. The results showed that PERK inhibitor LDN-0060609 has the highest activity at concentration of 25 μM, since it evoked a 65% inhibition of the eIF2α phosphorylation in HTM cells with activated ER stress conditions. What is more, used of the analyzed PERK inhibitor resulted in inhibition of the eIF2α phosphorylation at a similar level to the commercially available inhibitor GSK2606414 (GlaxoSmithKline), that constituted an additional control and triggered 70% inhibition of the eIF2α phosphorylation in HTM cells. No statistically significant differences were found in the inhibition of the eIF2α phosphorylation between the commercially available inhibitor GSK2606414 and the investigated PERK inhibitor LDN-0060609 at the concentration of 25 μM and 50 μM. Moreover, there were no statistically significant differences between the concentrations of 25 μM and 50 μM of the PERK inhibitor LDN-0060609 (Figure 1).

### 2.2. Analysis of Gene Expression of the Pro-Apoptotic ER Stress Markers

**Figure 2:**
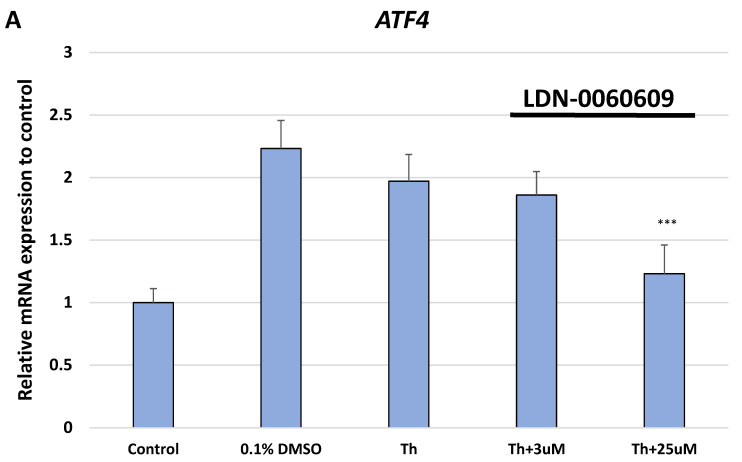

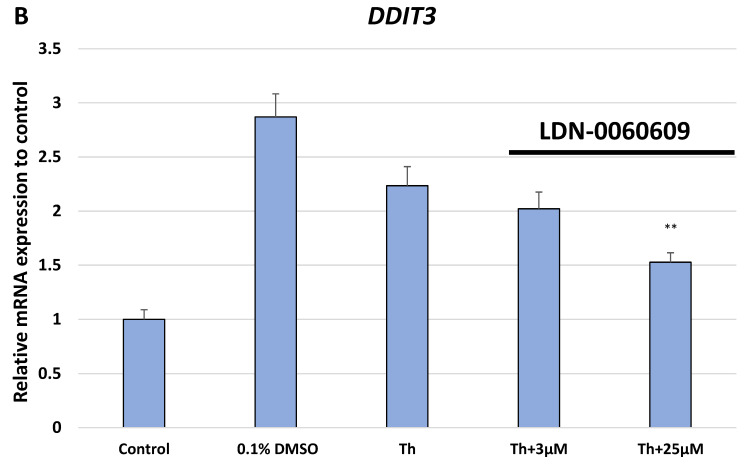

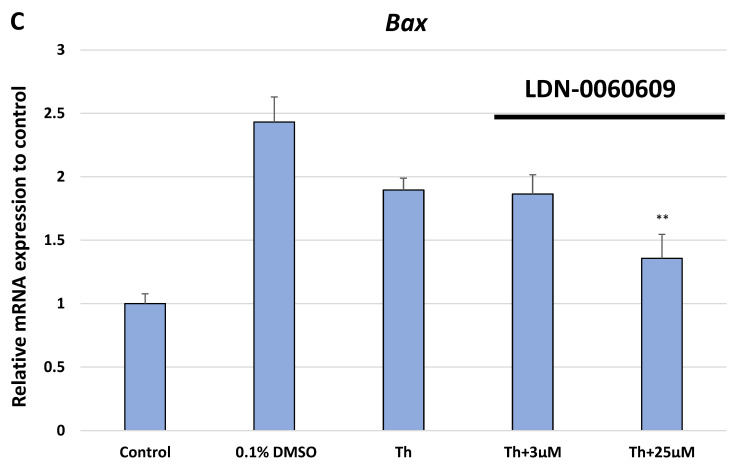
Analysis of the expression profile of the ER stress marker genes: *ATF4* (**A**), *DDIT3* (**B**) and *Bax* (**C**) in HTM cells with Th-induced ER stress conditions or treated with both Th and PERK inhibitor LDN-0060609. Analysis was performed using the TaqMan gene expression assay. Each of the statistical analyses in the individual experiments was based on the results of three independent tests. Data are expressed as mean ± SE (*n* = 3). On the graphs, the differences were statistically significant as follows: ** *p* < 0.01, *** *p* < 0.001 versus Th. Control—untreated HTM cells; DMSO—dimethyl sulfoxide; Th—thapsigargin.

Analysis of the gene expression profile of the ER stress marker genes in HTM cells, with Th-induced ER stress conditions, treated with the PERK inhibitor LDN-0060609 at a concentration of 25 μM demonstrated a significant decrease of the pro-apoptotic genes expression such as *ATF4*, *DDIT3* encoding CHOP, and *Bax* as compared to only Th-treated HTM cells (Figure 2).

### 2.3. Analysis of the Cytotoxicity and Pharmacological Effect of the PERK Inhibitor LDN-0060609

**Figure 3:**
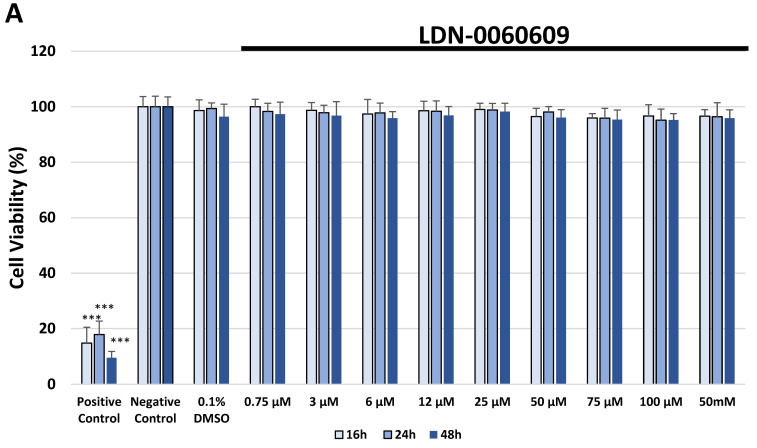

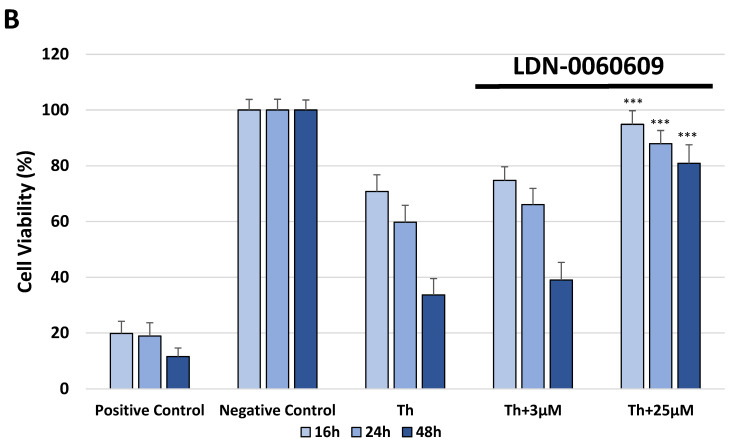
Analysis of cytotoxicity of the PERK inhibitor LDN-0060609 only (**A**) and after treatment of HTM cells with the Th and PERK inhibitor LDN-0060609 (**B**) by the XTT assay. Each of the statistical analysis in individual experiments was based on the results of three independent tests. Data are expressed as mean ± SE (*n* = 3). On the graphs, the differences were statistically significant as follows: *** *p* < 0.001 versus the negative control (**A**) and versus Th (**B**). DMSO—dimethyl sulfoxide; Th—thapsigargin.

**Figure 4:**
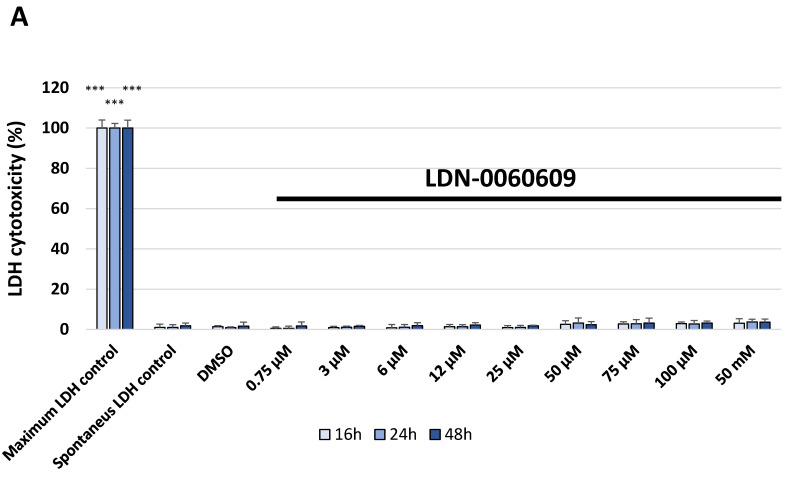

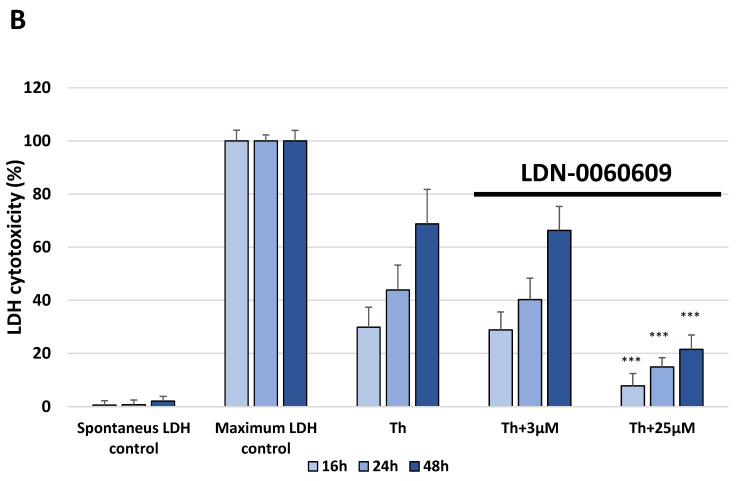
Analysis of cytotoxicity of the PERK inhibitor LDN-0060609 only (**A**) and after treatment of HTM cells with the Th and PERK inhibitor LDN-0060609 (**B**) by the lactate dehydrogenase (LDH) assay. Each of the statistical analyses in the individual experiments was based on the results of three independent tests. Data are expressed as mean ± SE (*n* = 3). On the graphs, the differences were statistically significant as follows: *** *p* < 0.001 versus the spontaneous LDH control (**A**) and Th (**B**). DMSO—dimethyl sulfoxide; Th—thapsigargin.

**Figure 5:**
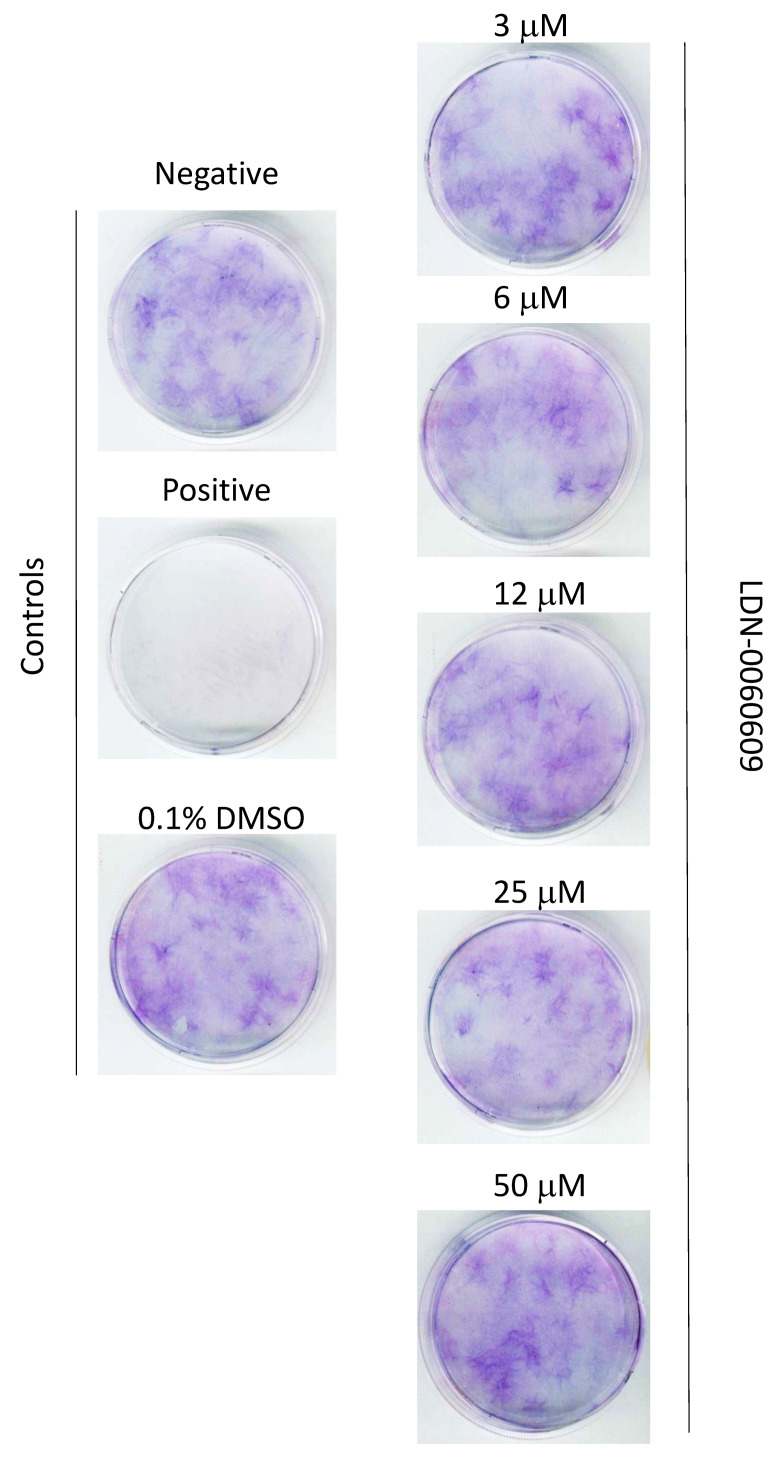
Cell survival assay on HTM cells treated with the PERK inhibitor LDN-0060609 at indicated concentrations (3–50 µM). DMSO—dimethyl sulfoxide.

The cellular toxicity of the investigated PERK inhibitor LDN-0060609 was measured in the HTM cell line by the 2,3-Bis-(2-Methoxy-4-Nitro-5-Sulfophenyl)-2H-Tetrazolium-5-Carboxanilide (XTT) assay, lactate dehydrogenase (LDH) assay and cell survival assay with Giemsa staining. No significant cytotoxic effect was observed in HTM cells at any applied concentration of the PERK inhibitor LDN-0060609 or incubation time. Moreover, 0.1% DMSO, that is used as a solvent for the PERK inhibitor, did not cause a significant cytotoxic effect toward HTM cells after 16, 24 and 48 h incubation in XTT and LDH assays as well as after 48 h in cell survival assay with Giemsa staining (Figure 3A, Figure 4A and Figure 5).

Effect of the investigated LDN-0060609 compound on cell viability was also evaluated, both by XTT and LDH assays, in the HTM cells with Th-induced ER stress conditions and treated with the PERK inhibitor LDN-0060609 at the concentrations of 3 μM and 25 μM. The results obtained from both performed tests demonstrated a significant decrease of the cell viability of the HTM cells treated with Th, as compared to negative control at all incubation times. However, after treatment of the HTM cells, with induced ER stress conditions, with the 25 μM PERK inhibitor LDN-0060609, we determined a significant increase in the HTM cells viability as compared to Th only treated cells at all incubation times (Figure 3B and Figure 4B).

### 2.4. Analysis of the Genotoxicity and Pharmacological Effect of the PERK Inhibitor LDN-0060609

**Figure 6:**
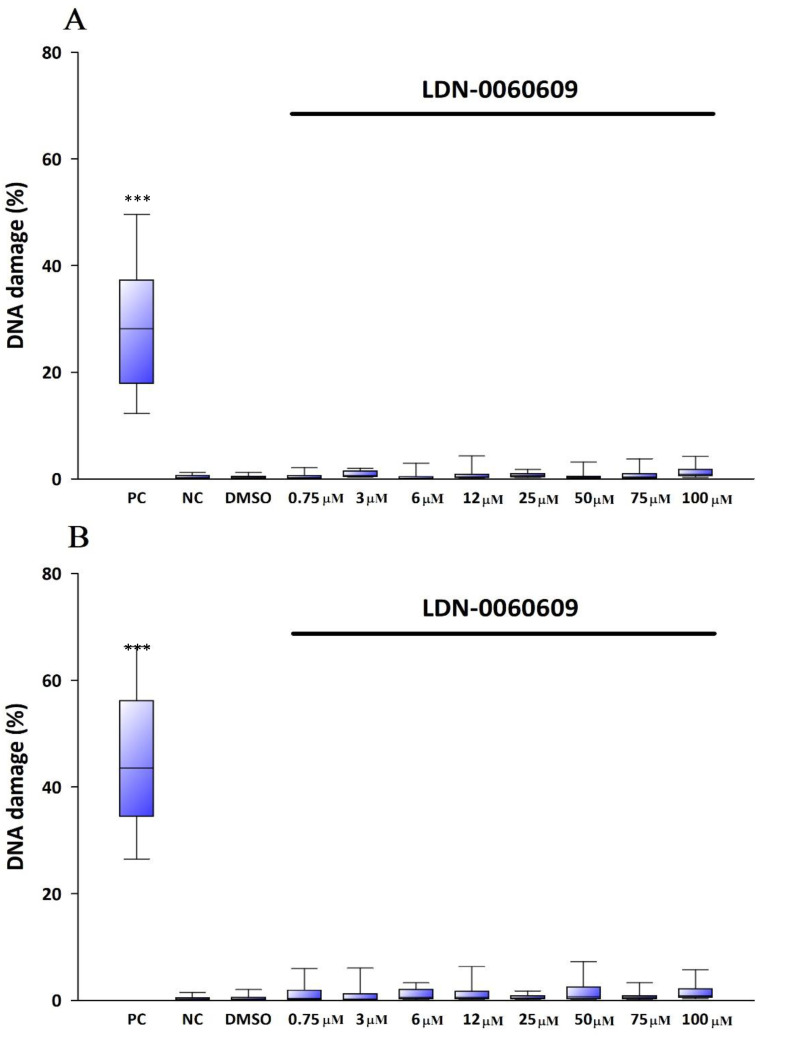

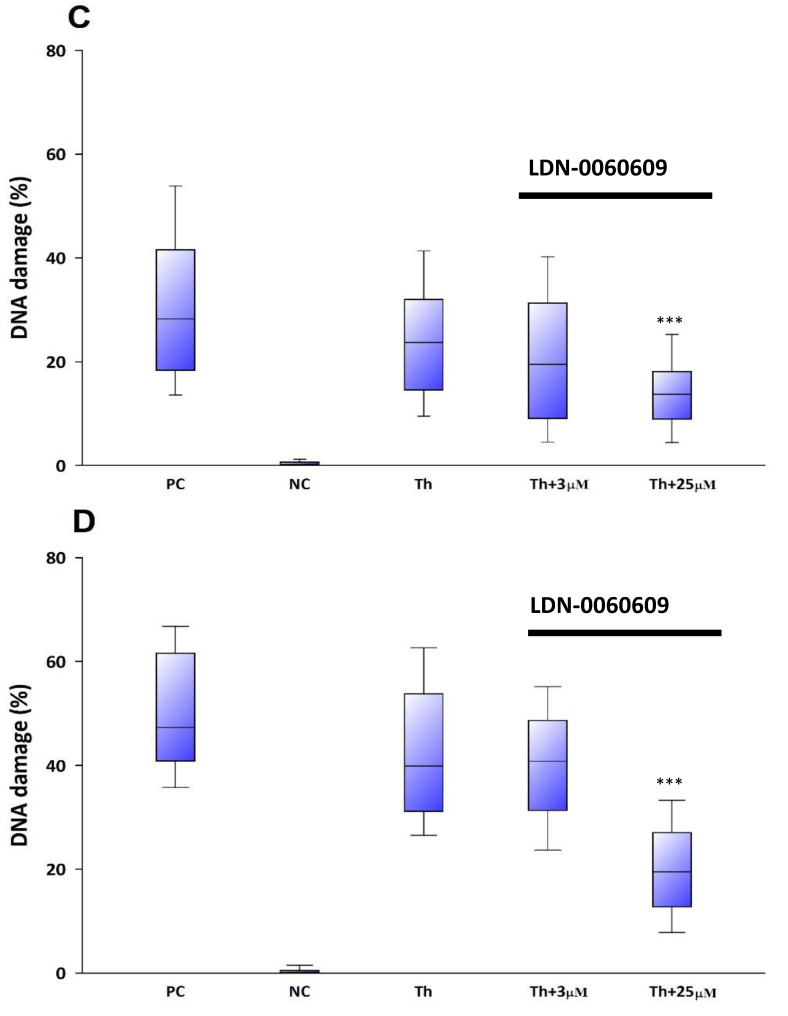
Analysis of genotoxicity in HTM cells treated only with the PERK inhibitor LDN-0060609 after 24 h (**A**) and 48 h (**B**) and HTM cells treated with Th and PERK inhibitor LDN-0060609 after 24 h (**C**) and 48 h (**D**) by the comet assay. Each of the statistical analyses in the individual experiments was based on the results of three independent tests. The value of cells scored for each individual was 100. Box plots show median, 25–75 percentile, minimum and maximum values. On the graphs, the differences were statistically significant as follows: *** *p* < 0.001 versus the negative control (NC) (**A**,**B**) and versus Th (**C**,**D**). PC—positive control; DMSO—dimethyl sulfoxide; Th—thapsigargin.

The alkaline version of the comet assay was used to evaluate the level of DNA damage induced by the investigated PERK inhibitor. The alkaline comet assay detects oxidative DNA damage, single- and double-stranded breaks and presence of alkaline-labile sites. The amount of DNA damage was assessed based on the percentage of DNA in the comet tail. Obtained results showed that PERK inhibitor LDN-0060609 did not induce a significant DNA damage within HTM cells at any used concentration after 24 h (Figure 6A) and 48 h (Figure 6B) incubation. Moreover, a solvent for the PERK inhibitor, 0.1% DMSO, did not evoke a significant genotoxic effect toward HTM cells after 24 h (Figure 6A) and 48 h (Figure 6B) incubation.

The significant increase in DNA damage in HTM cells, as compared to negative control, was observed after their treatment with Th both for 24 h (Figure 6C) and 48 h (Figure 6D). By contrast, treatment of HTM cells, with Th-induced ER stress conditions, with the 25 µM PERK inhibitor LDN-0060609 resulted in a significant reduction of DNA damage as compared to only Th-treated cells both after 24 h (Figure 6C) and 48 h incubation (Figure 6D).

### 2.5. Effect of the PERK Inhibitor LDN-0060609 on Morphological Changes in HTM Cells under ER Stress Conditions

**Figure 7:**
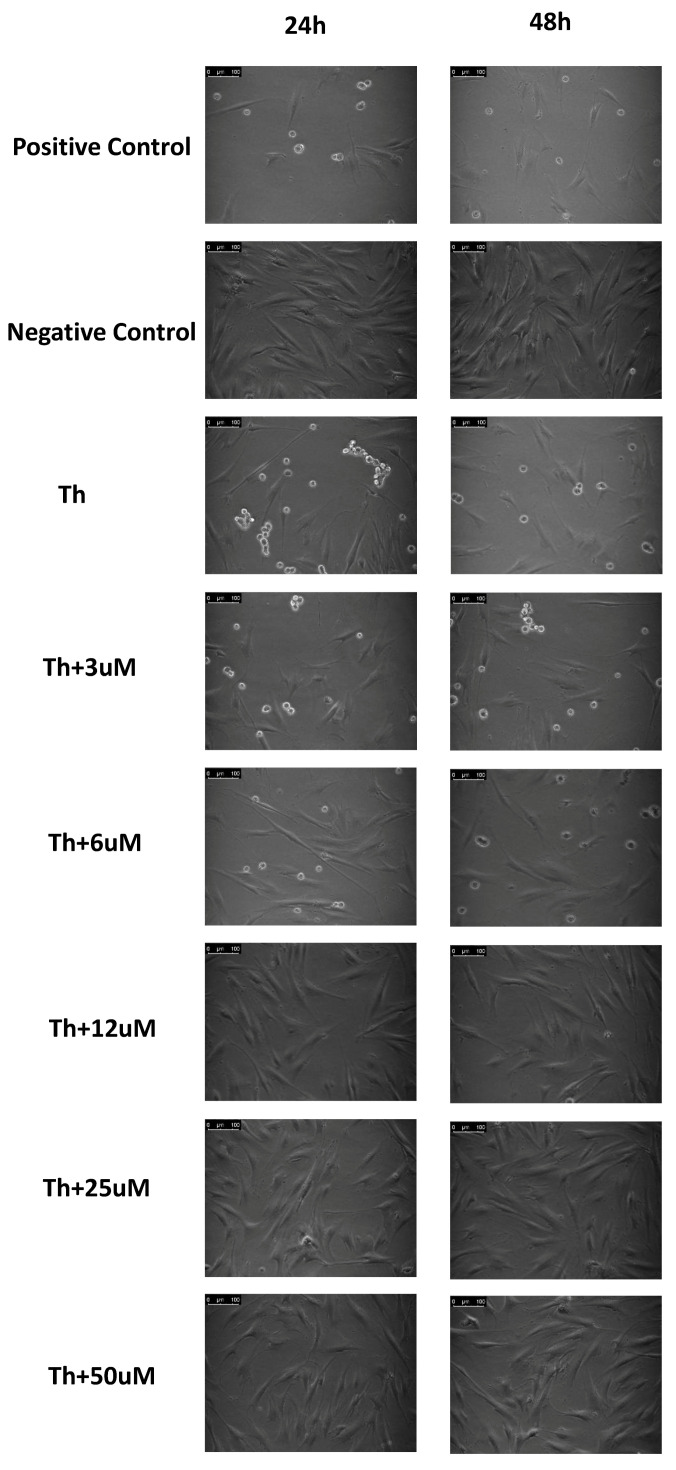
Effect of the PERK inhibitor LDN-0060609 on morphology of HTM cells, with induced ER stress conditions, after 24 h and 48 h incubation. Th—thapsigargin.

The effect of the PERK inhibitor LDN-0060609 on morphological changes in HTM cells with induced ER stress conditions was assessed by the phase-contrast microscopy. The normal morphology of the HTM cells was lost after their treatment with Th both for 24 h and 48 h as compared to the untreated cells (negative control). HTM cells treated with Th displayed morphological changes similar to HTM cells treated with 100% DMSO (positive control). Thus, treatment with Th induced ER stress conditions within HTM cells, since their confluency was significantly decreased and Th-treated cells were shrunk in size as compared to negative control. Loosening of cell-cell contact and the acquisition of a more spindle-shaped morphology after stimulation of HTM cells with Th were observed. Moreover, Th-treated HTM cells lost their capacity to attach to the culture vessels and became suspended. However, treatment of HTM cells with Th-induced ER stress conditions with the investigated PERK inhibitor LDN-0060609 both for 24 h and 48 h undid the negative effect of Th in a concentration-dependent manner. With the increasing concentration of the PERK inhibitor LDN-0060609, an increase in the proliferation of HTM cells, their return to normal morphology and a decreased ability to detach from the culture vessel were observed. No significant differences in the confluence and morphology of HTM cells, under ER stress conditions, were observed between those treated with 25 µM and 50 µM concentrations of the PERK inhibitor LDN-0060609 both after 24 h and 48 h incubation (Figure 7).

### 2.6. Evaluation of the Caspase-3 Activity in HTM Cells Treated Only with PERK Inhibitor LDN-0060609 or Thapsigargin and PERK Inhibitor LDN-0060609

**Figure 8:**
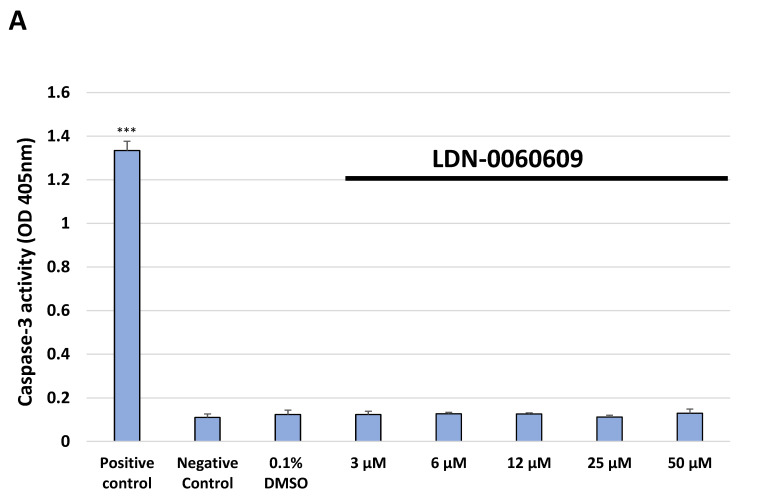

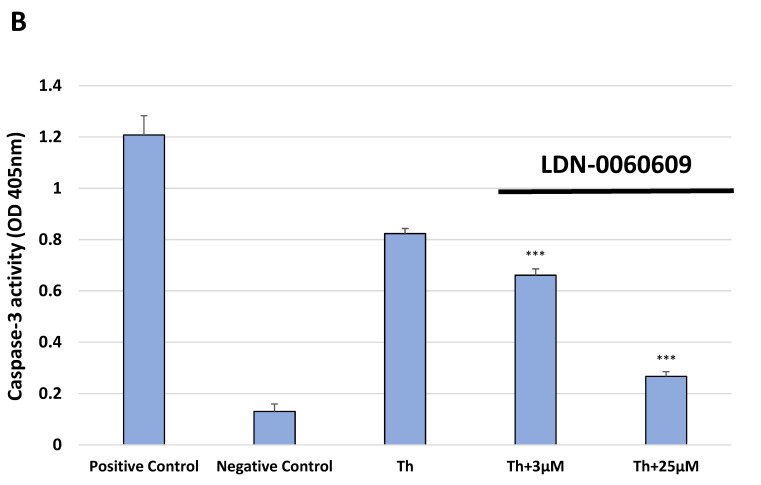
Analysis of apoptosis of the HTM cells treated with PERK inhibitor LDN-0060609 only (**A**) or with Th and PERK inhibitor LDN-0060609 (**B**) by the caspase-3 activity assay. Each of the statistical analyses in the individual experiments was based on the results of three independent tests. Data are expressed as mean ± SE (*n* = 3). On the graphs, the differences were statistically significant as follows: *** *p* < 0.001 versus the negative control (**A**) and versus Th (**B**). DMSO—dimethyl sulfoxide; Th—thapsigargin.

To assess an ability to induce apoptosis in HTM cells after their exposure to the PERK inhibitor LDN-0060609, a colorimetric caspase-3 assay was used. The results demonstrated that treatment of HTM cells with 1 µM staurosporine for 16 h significantly increased the level of caspase-3 activity in comparison with the control cells incubated only with the complete medium. There was no significant increase in the activity of caspase-3 after 24 h incubation of HTM cells with the investigated PERK inhibitor LDN-0060609 at any used concentration. Moreover, 0.1% DMSO, used as a solvent for the PERK inhibitor, did not result in a significant activation of the caspase-3-dependent apoptosis in the investigated cell line after 24 h incubation (Figure 8A).

Caspase-3 activity was also evaluated in HTM cells with induced ER stress conditions, after their treatment with the PERK inhibitor LDN-0060609 at a concentration of 3 μM and 25 μM. Obtained results demonstrated a significant increase in the caspase 3-activity after treatment of HTM cells only with Th for 16 h as compared to the control cells, incubated for 24 h with the complete medium. However, following 24 h incubation of HTM cells with Th and the investigated LDN-0060609 compound at 25 μM, the caspase-3 activity was significantly decreased as compared to Th-treated HTM cells (Figure 8B).

### 2.7. Detection of Apoptosis by FITC Annexin V/PI Double Staining in HTM Cells Treated Only with PERK Inhibitor LDN-0060609 or Thapsigargin and PERK Inhibitor LDN-0060609

**Figure 9:**
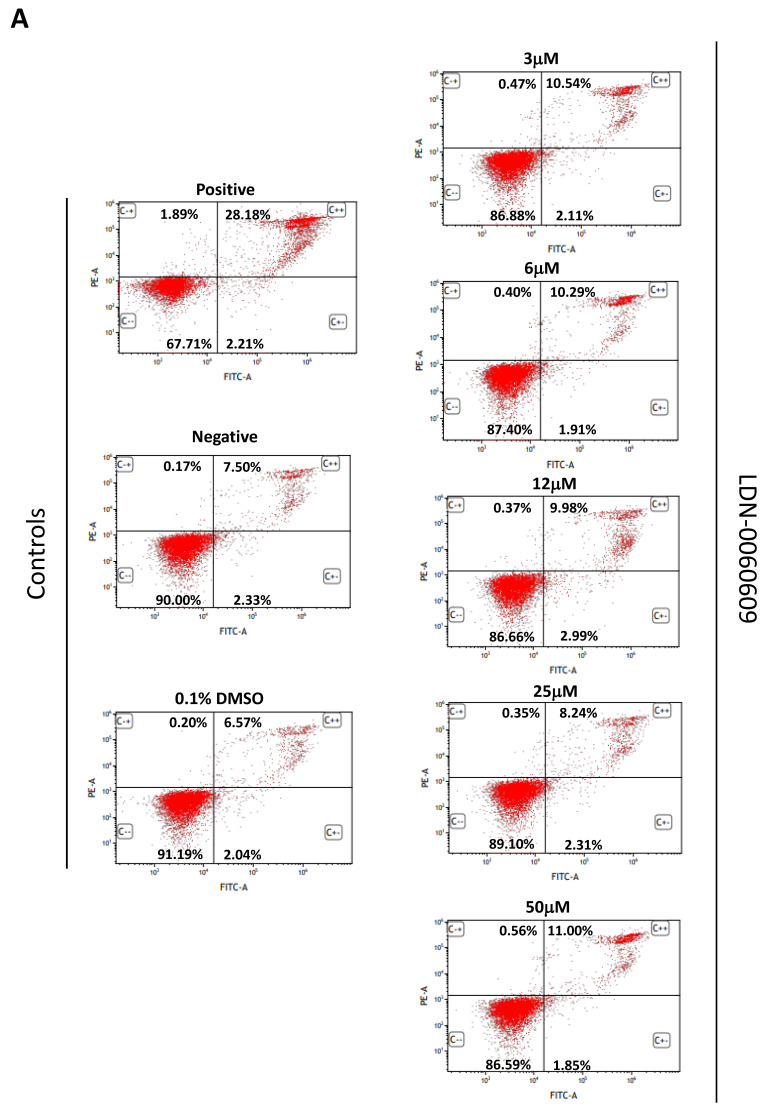

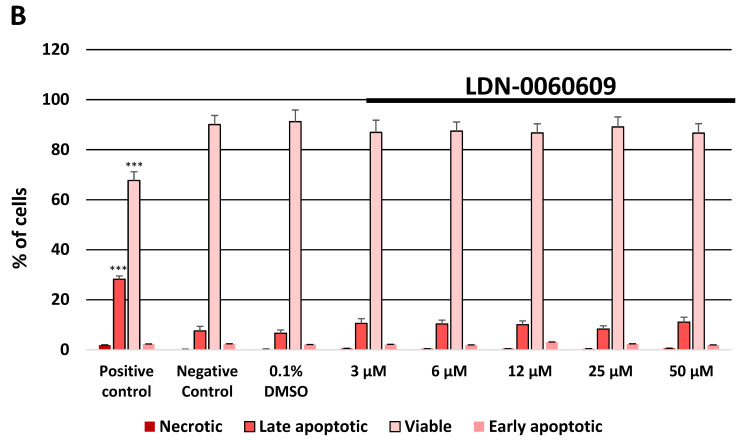

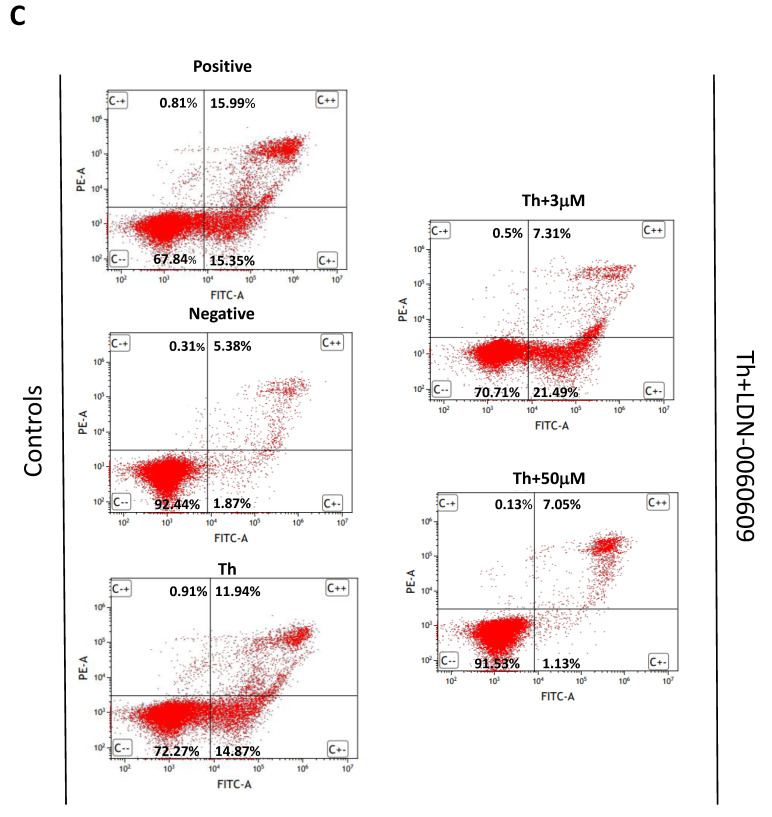

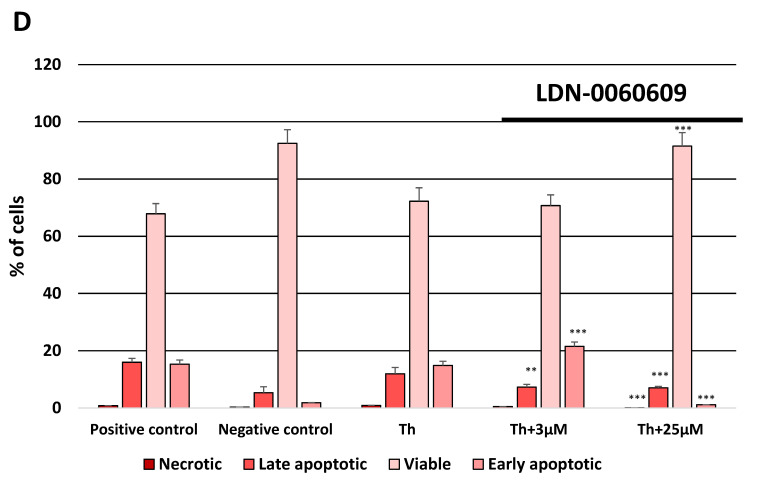
Analysis of apoptosis of the PERK inhibitor LDN-0060609 only (**A**,**B**) and after treatment of HTM cells with the Th and PERK inhibitor LDN-0060609 (**C**,**D**) by flow cytometric FITC annexin V/propidium iodide (PI) double staining. Dot plot graphs (**A**,**C**) and bar graphs (**B**,**D**), determined from dot plot graphs, indicate the percentage of viable (FITC annexin V negative, PI negative), early apoptotic (FITC annexin V positive, PI negative), late apoptotic (FITC annexin V positive, PI positive) and necrotic (FITC annexin V negative, PI positive) HTM cells. Each of the statistical analysis in individual experiments was based on the results of three independent tests. Data are expressed as mean ± SE (n = *3*). On the bar graphs, the differences were statistically significant as follows: ** *p* < 0.01, *** *p* < 0.001 versus the negative control (**B**) and versus Th (**D**). DMSO—dimethyl sulfoxide; Th—thapsigargin.

Activation of apoptosis in HTM cells exposed to the investigated PERK inhibitor LDN-0060609 at a concentration range of 3–50 μM for 24 h was analyzed with FITC annexin V/propidium iodide (PI) double staining by flow cytometry. After treatment with 1 µM staurosporine for 16 h, a significant number of HTM cells underwent apoptosis as compared to control cells, incubated for 24 h with the complete medium. The obtained results demonstrated that PERK inhibitor LDN-0060609 did not evoke a significant activation of apoptotic cell death in the investigated cell line at any used concentration and incubation time. Furthermore, 0.1% DMSO, used as a solvent for the PERK inhibitor, did not trigger a significant activation of apoptotic cell death of HTM cells after a 24 h incubation. Additionally, there was no increase in the level of necrotic cells after 24 h exposure to 0.1% DMSO or the analyzed PERK inhibitor at any used concentration (Figure 9A).

To assess activation of apoptosis in HTM cells with induced ER stress conditions and treated with the PERK inhibitor LDN-0060609 at a concentration of 3 μM and 25 μM, a flow cytometry using FITC annexin V/propidium iodide (PI) double staining was also used. Obtained results demonstrated that 31.34% of HTM cells treated with staurosporine (positive control) for 16 h underwent apoptosis, whereas there were 26.81% of HTM apoptotic cells treated only with Th for 24 h. Interestingly, following 24 h incubation of HTM cells, with induced ER stress conditions with the investigated PERK inhibitor LDN-0060609 at a concentration of 25 μM, the percentage of apoptotic HTM cells, as compared to the positive control, was significantly decreased up to 8.18% (Figure 9B).

### 2.8. Evaluation of the Cell Cycle Progression by PI Staining in HTM Cells Exposed to PERK Inhibitor LDN-0060609 or Thapsigargin and PERK Inhibitor LDN-0060609

**Figure 10:**
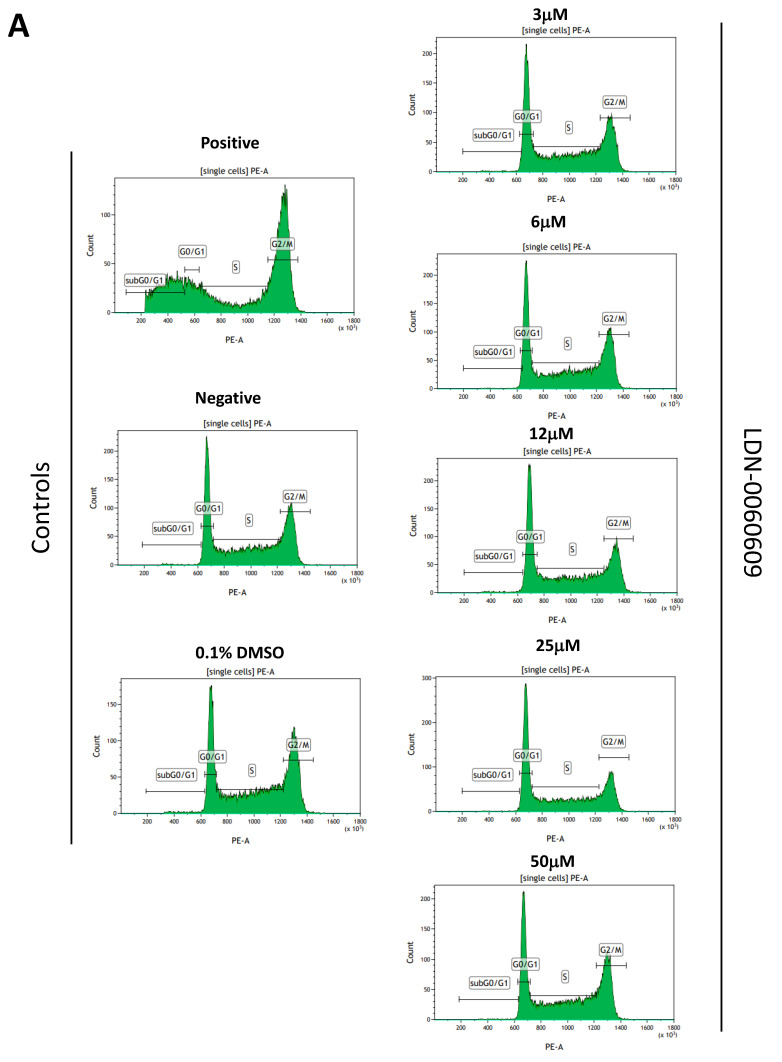

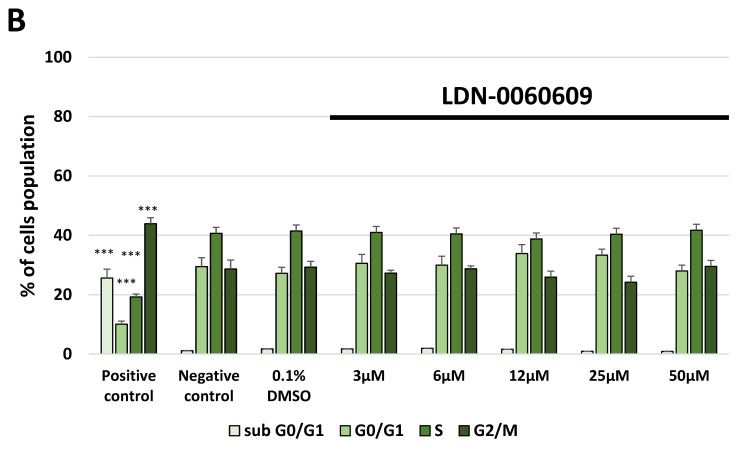

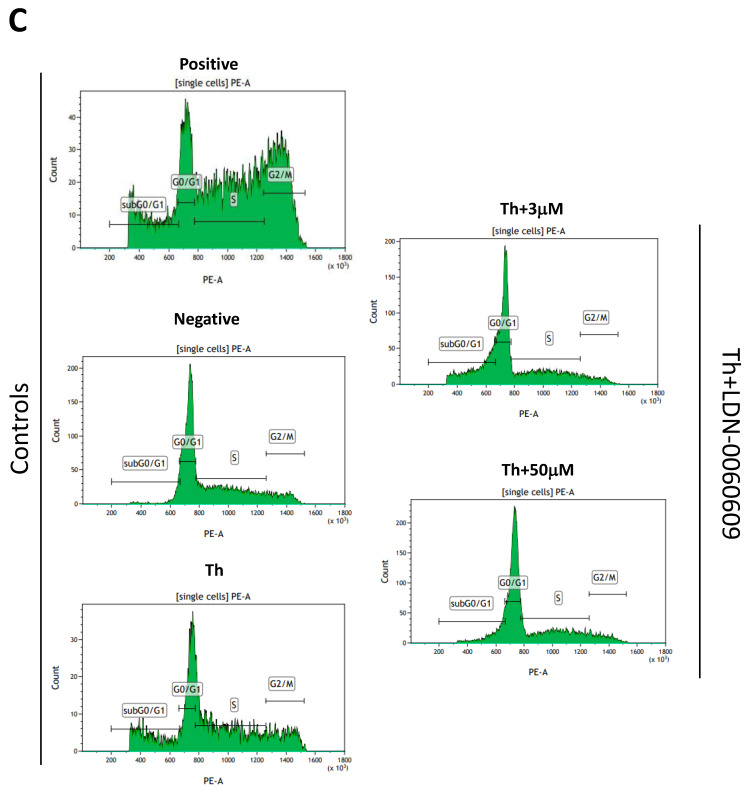

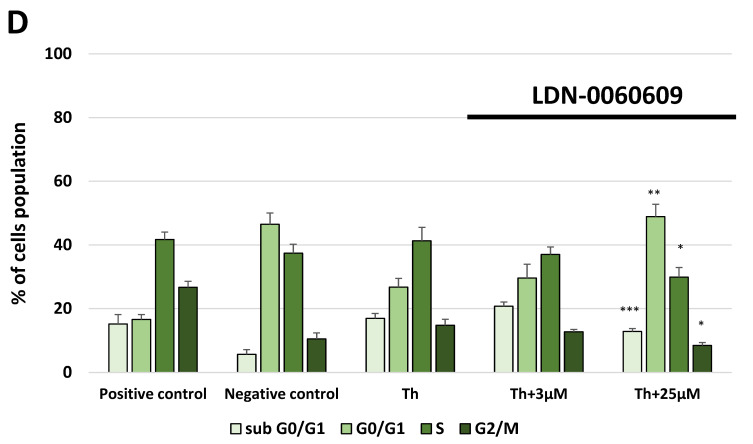
Analysis of cell cycle progression of HTM cells treated with the PERK inhibitor LDN-0060609 only (**A**,**B**) and after treatment of HTM cells with the Th and PERK inhibitor LDN-0060609 (**C**,**D**) by flow cytometric propidium iodide (PI) staining. DNA content histograms (**A**,**C**) and cell cycle distribution bar graph determined from DNA content histograms (**B**,**D**). Each of the statistical analysis in individual experiments was based on the results of three independent tests. Data are expressed as mean ± SE (*n* = 3). On the graphs, the differences were statistically significant as follows: * *p* < 0.05, ** *p* < 0.01, *** *p* < 0.001 versus the negative control (**B**) and versus Th (**D**). DMSO—dimethyl sulfoxide; Th—thapsigargin.

The cell cycle distribution in PI-stained HTM cells previously exposed to the PERK inhibitor LDN-0060609 was analyzed by flow cytometry. Results obtained showed that there was no effect of the investigated PERK inhibitor on the course of cell cycle progression of HTM cells. Arrest in the G2/M phase of the cell cycle was observed only in HTM cells treated with nocodazole at 1 μM for 16 h, that constituted a positive control. Results obtained showed that there was no significant differences between the proportion of cells in G0/G1, S and G2/M phases between the control cells, cultured only in a complete medium, and cells treated with the PERK inhibitor LDN-0060609 for 24 h at all concentrations used, and therefore no cell cycle arrest was observed. Additionally, results showed that there was no effect of the 0.1% DMSO on the course of cell cycle of HTM cells after 24 h incubation (Figure 10A,B).

The cell cycle distribution was also evaluated via flow cytometry using the PI staining in HTM cells, with Th-induced ER stress conditions, after their treatment with the PERK inhibitor LDN-0060609 at the concentrations of 3 μM and 25 μM. Significant arrest in the G2/M phase of the cell cycle was demonstrated in the nocodazole-treated HTM cells (positive control) for 16 h as compared to the untreated cells (negative control). In addition, flow cytometric analysis of the cell cycle distribution of HTM cells treated only with Th demonstrated an increase in the mean of percentage of HTM cells in the G2/M phase of the cell cycle as compared to the negative control. However, following treatment of HTM cells with Th-induced ER stress conditions with the PERK inhibitor LDN-0060609 at a concentration of 25 μM for 24 h, we have noticed a significant decrease of the percentage of cells in G2/M as compared to only Th-treated HTM cells (Figure 10C,D).

## 3. Discussion

In the present research, the effectiveness of the small-molecule PERK inhibitor LDN-0060609 was evaluated in the cellular experimental model of glaucoma using HTM cells derived from the juxtacanalicular and corneoscleral regions of the human eye. In this study, we have demonstrated the response of HTM cells to the ER stress activator, Th, as well as the pharmacological effect of the small-molecule PERK inhibitor LDN-0060609 in the cellular model of glaucoma. Specifically, HTM cells with Th-induced ER stress conditions were used as a cellular model of POAG. The results obtained have shown that treatment of HTM cells with Th markedly elevates phosphorylation of the main substrate of PERK, that is eIF2α, which is indicative of activation of the ER stress conditions. Interestingly, investigated PERK inhibitor LDN-0060609 triggered significant inhibition of the eIF2α phosphorylation in HTM cells with induced ER stress conditions. In addition, our research also includes the analysis of the expression of genes encoding other markers of the ER stress-dependent UPR signaling pathway, namely *ATF4*, *DDIT3* and *Bax*, after treatment of HTM cells with the investigated PERK inhibitor. It has been demonstrated that treatment of HTM cells, with induced ER stress conditions, with the PERK inhibitor LDN-0060609 resulted in significant decrease of the expression of pro-apoptotic genes such as *ATF4*, *DDIT3* and *Bax.* No cytotoxic or genotoxic effect was observed in HTM cells at any used concentration of the PERK inhibitor LDN-0060609 and at any time of incubation. However, the pharmacological effect of the PERK inhibitor LDN-0060609 was confirmed in the cellular model of POAG, as LDN-0060609 compound markedly undid the negative effect of Th-induced ER stress in HTM cells via increasing their viability and reducing DNA damage. Interestingly, PERK inhibitor LDN-0060609 also effectively decreased the level of ER stress in Th-treated HTM cells, as it increased cell proliferation and restored normal cell morphology. Furthermore, results from the present study have demonstrated that investigated PERK inhibitor did not provoke a significant activation of apoptotic cell death as well as it had no effect on the course of cell cycle progression in HTM cell line. However, treatment of HTM cells, with induced ER stress conditions, with the PERK inhibitor LDN-0060609 triggered a significant decrease in the percentage of apoptotic cells, as well as a decrease in the percentage of cells at G2/M phase of the cell cycle, as compared to only Th-treated HTM cells. Thus, these findings indicate that small-molecule PERK inhibitor LDN-0060609 may contribute to the significant reduction of negative effects associated with ER stress, mainly cell apoptosis, that plays a key role in glaucoma pathogenesis.

Interestingly, results obtained in this study confirmed a high potency of the small-molecule PERK inhibitor LDN-0060609 in the treatment of neurodegenerative disease such as POAG. In our previous study, treatment of phenotype type 1 rat normal astrocytes derived from diencephalon (DI TNC1) with investigated LDN-0060609 compound triggered a significant inhibition of the eIF2α phosphorylation under Th-induced ER stress conditions. Moreover, results obtained have demonstrated that analyzed PERK inhibitor LDN-0060609 is non-cytotoxic toward DI TNC1 cells as well as it did not induce significant apoptosis of DI TNC1 cells and had no effect on their cell cycle progression [33]. Also, research conducted on mouse neurons CATH.a demonstrated that LDN-0060609 compound effectively inhibits ER stress-mediated apoptotic cell death via reduction of the CHOP protein level under ER stress conditions and does not evoke a cytotoxic effect toward CATH.a cells [42]. It has also been demonstrated that PERK inhibitor LDN-0060609 evoked a significant inhibition of eIF2α phosphorylation in neuroblastoma SH-SY5Y cell line with Th-induced ER stress conditions. In addition, LDN-0060609 compound did not induce a cytotoxic effect toward SH-SY5Y cells [43]. The results obtained are promising and shed light on the potential usefulness of the investigated compound in neuroprotection of RGCs in glaucoma-induced optic nerve damage, and this aspect should be further evaluated.

Emerging evidence suggests that reactive oxygen species (ROS), free radicals, oxidative and reductive stress are directly correlated with POAG pathogenesis. It has been reported that oxidative DNA damage was markedly increased in the ocular epithelium regulating AH outflow, namely the TM, of glaucomatous patients. Interestingly, the following research findings supported the role of ROS in glaucoma pathogenesis: resistance to AH outflow is increased by hydrogen peroxide by inducing TM degeneration; TM has antioxidant activities, primarily related to superoxide dismutase-catalase and glutathione pathways that are disturbed in glaucoma patients [44]. Furthermore, it has been demonstrated that both IOP increase and severity of visual-field damage in glaucoma patients are directly related to the amount of oxidative DNA damage of TM cells. Oxidative stress may induce human TM degeneration, favoring an IOP increase, thus promoting the POAG pathogenetic cascade [45]. Besides, study by Erdurmuş et al. has demonstrated that decreased antioxidant defense and increased oxidative stress may lead to POAG development and progression [46]. Zanon-Moreno et al. have reported that glaucomatous human eyes were characterized by a significant increase in oxidative status and decreased antioxidant activity in the AH, thus oxidative stress may play an important role in the POAG pathogenesis [47]. Due to the fact that glaucoma-related cell apoptosis may be activated via the oxidative stress-dependent manipulation of intracellular redox status, it would be beneficial if a novel therapy against glaucomatous neurodegeneration is based on these findings [48]. Interestingly, based on the radiolysis studies, it has been demonstrated that investigated PERK inhibitor LDN-0060609 (named as AMD) may play an important role in the scavenging the excessive amount of free radicals [41]. Thereby, results obtained in our present study as well as other research data have confirmed that LDN-0060609 compound constitutes a novel molecule that may effectively inhibit the PERK-dependent UPR signaling pathway responsible for neurodegenerative processes as well as constitutes an effective free radical scavenger.

Glaucoma, that constitutes a group of optic neuropathies, is closely associated with rapid RGCs loss, damage to the optic nerve and subsequent irreversible loss of vision. It has been demonstrated that the major factor directly leading to POAG pathogenesis constitutes increased IOP. In glaucomatous individuals, the flow of AH is disrupted, that results in the markedly increased IOP. Above-mentioned perturbations contribute to the development of degenerative changes as well as loss of RGCs [49]. In the present study, the HTM cell line was used as a cellular experimental model of POAG. Trabecular meshwork (TM) cells play a significant role in the maintenance of the normal AH outflow system. Loss of the vitality and functions of the TM cells may directly lead to the obstruction of the AH outflow and subsequently to the IOP elevation. Interestingly, it has been reported that perturbations in the passage of AH via the TM have been attributed primarily to the innermost region of the TM, that is juxtacanalicular region [50,51]. There are several lines of evidence that injury or death of TM cells is strictly related to the POAG pathogenesis. Interestingly, Baleriola et al. have demonstrated that the tendency to accumulate more apoptotic TM cells was higher in patients with POAG than in those with primary ACG [52]. It has been reported that structural alterations in the TM in the eyes of POAG patients, as compared to non-glaucomatous individuals, are strictly correlated with the significant decrease in cellularity [53], increase in the extracellular matrix (ECM) and the presence of plaque material in the juxtacanalicular tissue. Hence, above-mentioned alterations in the TM may trigger a significant perturbations in the outflow resistance [54]. Thereby, based on the literature data, TM cells, especially those from the juxtacanalicular region, provide an invaluable tool to study the functional control of TM as well as they provide an excellent in vitro cellular model, the main aim of which is to develop more efficient strategies to treat glaucoma.

The major mechanism directly responsible for reduction of TM cell population in glaucomatous and normal individuals still remains unknown, whereas various potential mechanisms are suggested including wear-and-tear, phagocytosis, cell migration, and cell death. Not only the correlation between TM cell loss and the apoptosis in POAG individuals, but also the mechanisms underlying the apoptosis in the TM require further characterization. It has been suggested that intense phagocytic activity in TM cells may trigger apoptotic cell death. Moreover, glaucoma itself may also elicit apoptosis of TM cells via induction of the mechanical stress and strain to the axons of the optic nerve or trabecular hypoperfusion. Excessive oxidative stress may as well trigger cell loss or alterations in the TM cells’ function [52]. It has also been proposed that disruption in TM functioning is often modulated by integrins, the receptor proteins which provide connections between intracellular cytoskeleton and ECM proteins. Integrins-mediated signaling events induce changes in the actin cytoskeleton of the TM, increase in the TM outflow resistance, IOP elevation, neuroinflammation and re-modeling within the optic nerve head [55]. Interestingly, there is ample evidence that ER stress-dependent apoptosis may contribute to the development and further progression of numerous human diseases, including ocular diseases [56]. The molecular etiology of glaucoma is still not fully elucidated, whereas the newest data has provided evidence that ER stress and subsequent activation of the UPR signaling pathway may act as a major contributor to POAG pathogenesis at the molecular level [57,58,59].

ER stress may be induced by numerous factors, and the one strictly associated with glaucoma pathogenesis may constitute the mutation in *myocilin* (*MYOC*) gene, as one of the major genetic factors responsible for POAG development and progression. Multiple research data has confirmed the role of *MYOC* missense mutations in the activation of ER stress and the UPR signaling pathway, indicating their direct correlation with glaucoma pathogenesis [60,61,62]. As the mutant MYOC has a lower solubility and higher ability to aggregate as compared to the physiological form of MYOC, this may constitute one of the major causes of ER stress activation [63]. In fact, it has been confirmed that disease-causing *MYOC* mutations trigger reduction of MYOC solubility, that promotes the formation of secretion-incompetent aggregates within the ER lumen [64]. This event is specific for glaucoma, as myocilin amyloids are the only known form of pathogenic proteins that accumulate within the ER of TM cells, and TM cells are particularly vulnerable to myocilin toxicity. In addition, such myocilin inclusion bodies are found in about 70–80% of all glaucoma cases [65]. Although the detailed pathological mechanisms directly responsible for the disturbances in the AH outflow within TM and increased IOP are still unknown, the newest data has demonstrated that aggregation of abrogated proteins in the ER lumen may generate ER stress conditions, IOP elevation and subsequent damage of the TM, that is closely correlated with glaucoma pathogenesis [66]. One of the suggested underlying mechanisms is the mutant myocilin-induced abnormal accumulation of ECM proteins in the TM, that contributes to impediment of AH outflow and subsequent IOP elevation. Presence of both ECM and myocilin intracellular aggregates is related to induction of the ER stress markers like GRP78, PERK and CHOP [67,68]. Furthermore, whilst ER autophagy (reticulophagy) is essential for myocilin clearance, it has been found that ER-related chaperone glucose-regulated protein 94 (GRP94) specifically recognizes the mutated form of myocilin and targets it for ER-associated degradation (ERAD), which is a less effective way for degradation of misfolded myocilin [69]. As the mutant and damaged proteins like MYOC cannot be removed when the ER stress is activated, this in turn triggers TM cell impairment [57,70]. Interestingly, mutated MYOC induces the overexpression of GRP78 chaperones as well as the protein disulfide isomerase, which triggers perturbations in TM cellular morphology and normal cell proliferation, that may play a pivotal role in glaucoma pathogenesis [57].

Furthermore, there are other research data on direct correlation of glaucoma pathogenesis with activation of the ER stress-mediated UPR signaling pathway. For instance, it has been shown that apoptosis of RGCs may occur as a cellular response to the ER stress conditions, as the levels of marker proteins of the UPR signaling pathway, such as GRP78, p-eIF2α and CHOP, have been significantly elevated in RGCs after induction of ER stress conditions via tunicamycin [16]. Also, study by Doh et al. has demonstrated that apoptosis of the RGCs may occur in the ER stress-dependent manner, since the GRP78, p-PERK and p-eIF2 levels, upon IOP elevation, were significantly increased during the early stage of the UPR signaling pathway activation to protect RGCs against apoptosis. Interestingly, the expression of CHOP, the major UPR marker of apoptosis, was markedly increased when the IOP elevation was prolonged. Above-mentioned molecular event results in the RGCs apoptosis, that confirms a direct association of ER stress and activation of the PERK-dependent UPR signaling pathway with cell death during progression of chronic glaucoma [17]. Currently, glucocorticoid-induced glaucoma pathology is not fully understood, whereas study by Zode et al., in in vivo murine model of glucocorticoid-induced glaucoma, has demonstrated that ER stress results in glucocorticoid-induced ocular hypertension and suggested that reducing ER stress has potential as a therapeutic strategy for treating glucocorticoid-induced glaucoma [58]. Also, research by Wang et al. has confirmed a direct correlation between activation of the PERK-dependent UPR signaling pathway upon ER stress conditions and glaucoma pathogenesis. It has been demonstrated that ER stress triggers apoptotic cell death both in trabecular meshwork stem cells (TMSCs) and TM cells. Moreover, ER stress activation results in markedly increased expression of the UPR signaling pathways marker proteins, including GRP78, spliced X-box-binding protein-1 (sXBP1) and CHOP, in TM cells as compared to TMSCs. On the other hand, swollen ER and mitochondria were detected both in TMSCs and TM cells [18]. Besides, study by Peters et al. has confirmed that chronic ER stress is activated in human glaucomatous TM tissues and cells as well as suggested that ER stress-mediated signaling pathway may constitute a novel target for the developing disease-modifying glaucoma treatment strategies. Mentioned research has confirmed a significant elevation of chaperone proteins such as GRP78 and GRP94 in glaucomatous TM in comparison with normal TM. Moreover, chronic ER stress has been detected in glaucomatous TM, since a 3-fold increase of CHOP level has been noted. Also, increased expression of the major markers of ER stress, including GRP78, GRP94, ATF-4, ERO-1α, and CHOP, has been detected in glaucomatous TM tissue. Supposedly, if glaucomatous TM cells do not show splicing of X-box-binding protein-1 (XBP-1), a marker of the IRE-1-mediated UPR signaling pathway, it may be PERK, the second major ER stress transducer, that regulates the apoptotic TM cells death in CHOP-dependent mechanism [59]. However, study by Yang et al. has reported that manipulation of the UPR signaling pathways in opposite directions, namely inhibition of the PERK/eIF2α/CHOP arm of the UPR signaling pathway and, on the other hand, activation of the IRE1α-XBP-1 UPR signaling pathway, promotes both RGCs axons and somata survival and preserves visual function. Thereby, ER stress constitutes a promising therapeutic target for glaucoma and potentially other types of neurodegeneration [19].

Although there are multiple research data confirming a pivotal role of the PERK-dependent UPR signaling pathway in POAG pathogenesis, only a few studies have demonstrated that small-molecule PERK inhibitors may constitute a novel treatment strategy for glaucoma. Study by Wang et al. has shown that commercially available PERK inhibitor GSK2606414 significantly reduced cell survival rates, thereby enhanced apoptotic cell death both in TM cells and TMSCs with activated ER stress conditions via tunicamycin treatment. However, in Salubrinal-treated TM cells and TMSCs, with tunicamycin-induced ER stress conditions, an increased cell viability has been noted. Besides, TMSCs were less sensitive to the GSK2606414, but they were more sensitive to Salubrinal in comparison with TM cells. Based on the results obtained from this study, Wang et al. have reported that regulation of the PERK-mediated UPR signaling pathway in combination with stem cell therapy may contribute to the protection or regeneration of TM in glaucoma individuals [18]. Although a neuroprotective effect of the PERK inhibitor GSK2606414 has been confirmed in multiple models of neurodegenerative diseases [71,72,73], treatment with GSK2606414 triggered a cytotoxic effect, resulting in body weight loss and induction of hyperglycemia in vivo [71]. Moreover, it has been demonstrated that treatment with GSK2606414 in vivo elicited various side effects that might be directly linked to pancreatic toxicity [73]. To the best of our knowledge, apart from GSK2606414, no other specific inhibitors of the PERK-dependent signaling pathway have extensively been evaluated in preclinical glaucoma models, which brings novelty to our present research on the effect of LDN-0060609 on ER stressed HTM cells.

The other limitation of the in vitro studies conducted hitherto is the choice of experimental model, that is in most cases restricted to TM cells only. TM cells, also applied herein, are widely used in glaucoma research [74,75,76,77,78,79], although they are not the only tissue of the eye exposed to glaucoma-induced damage. Of note, specific molecular changes may also occur in the conjunctival epithelium and stroma within the filtering bleb after trabeculectomy, as evaluated by the optical coherence tomography (OCT) [80]. The results of such filtering surgery for glaucoma are significantly improved upon application of mitomycin C (MMC), which is an inhibitor of fibroblast proliferation and scarring processes. In study by Napoli et al., all primary trabeculectomies combined with MMC application were successful, which was also associated with OCT parameters like cystoid pattern and a low reflectivity of the bleb. These findings are indicative of a key role of fibroblast activity and the degree of scarring on the effectiveness of glaucoma filtration surgery [81]. It would be therefore of benefit to evaluate the effectiveness of the compounds modulating ER stress in above-mentioned types of cells, as well as in the models based on surgery-treated glaucoma. As most of the studies are primarily focused on POAG, there is also lack of data on pharmacological modulation of the UPR in other, less common types of glaucoma, such as angle-closure or normal tension glaucoma; for this purpose, novel, specific experimental models should be developed.

Currently, glaucoma etiology is not fully elucidated as well as treatment strategies against glaucoma are only symptomatic, thus they cannot prevent vision loss. However, above-mentioned literature data and results obtained from the present study have confirmed that ER stress conditions and activation of the PERK-dependent UPR signaling pathway may be directly correlated with POAG pathogenesis at the molecular level. Thus, targeting of the components of the UPR signaling pathways via small-molecule inhibitors like LDN-0060609 may constitute an innovative, ground-breaking treatment strategy against POAG.

## 4. Materials and Methods

### 4.1. Identification of PERK Inhibitor LDN-0060609

Investigated, small-molecule PERK inhibitor LDN-0060609 was screened, well-characterized and provided for further analysis in collaboration with the Department of Biochemistry and Molecular Biology, Hollings Cancer Center, Medical University of South Carolina, Charleston, SC, USA according to the methods previously described by Pytel et al. (2014) [82]. The Laboratory for Drug Discovery in Neurodegeneration (LDDN) compound library was used for the high-throughput assay (inhibitor screening).

### 4.2. Cell Culture

All experiments were performed in an in vitro cellular model using commercially available HTM cells (6590) isolated from the juxtacanalicular and corneoscleral regions of the human eye. The cell line was purchased from the ScienCell Research Laboratories (San Diego, CA, USA). Cell culture was maintained under standard conditions (37 °C; 5% CO_2_; 95% humidity), according to the guidelines provided by the vendors. Cells were cultured in trabecular meshwork cell medium (TMCM) (ScienCell Research Laboratories, San Diego, CA, USA), that contains basic medium (BM) (ScienCell Research Laboratories, San Diego, CA, USA), 2% fetal bovine serum (FBS) (ScienCell Research Laboratories, San Diego, CA, USA), 1% trabecular meshwork cell growth supplement (TMCGS) (ScienCell Research Laboratories, San Diego, CA, USA) and 1% penicillin/streptomycin solution (P/S) (ScienCell Research Laboratories, San Diego ad, CA, USA). Cells were cultured on poly-L-lysine-coated T-75 culture vessels (2 μg/cm^2^). Cells were split every 3–4 days, when the culture reached 90–95% confluency, after exposure to 0.05% trypsin/EDTA solution (T/E) solution (ScienCell Research Laboratories, San Diego, CA, USA).

### 4.3. Western Blot Analysis

To define the cellular response of HTM cells, with induced ER stress conditions, to treatment with the investigated PERK inhibitory compound LDN-0060609, the PERK enzyme activity was evaluated by measurement of the level of phosphorylated form of the eIF2α, that constitutes a major PERK substrate, by the Western blot technique. The experiment was repeated three times with similar results. HTM cells on a poly-L-lysine-coated T-75 culture vessels, at a confluence of 90–100%, were pretreated for 1 h with the investigated PERK inhibitory compound at a concentration range of 3–50 μM, 0.1% DMSO (Sigma-Aldrich Corp., St. Louis, MO, USA) or commercially available inhibitor GSK2606414 at a concentration of 1 µM, and then treated with the Th (500 nM), as an ER stress inducer, for 2 h. Cells incubated for 2 h only with Th (500 nM) served as a positive control, whereas cells cultured only in complete medium were used as a negative control. After treatment, total proteins were extracted using the MinuteTM Total Protein Extraction Kit For Animal Cultured Cells and Tissues (Invent Biotechnologies, Inc., Plymouth, MN, USA). Total protein extraction was performed by native cell lysis buffer (SN-002) supplemented with Protease and Phosphatase Inhibitor Cocktail (Thermo Fisher Scientific Inc., Waltham, MA, USA). Protein concentrations were determined by performing a standard Bradford assay. Bovine serum albumin (BSA) was used as a protein standard. Next, 80 µg of protein from each sample were mixed with 2 X SDS-PAGE loading buffer (Bio-Rad Laboratories, Hercules, CA, USA), and boiled at 95 °C for 10 min. Samples were resolved by 10% sodium dodecyl sulfate-polyacrylamide gel electrophoresis (SDS-PAGE) and then proteins were transferred onto a microporous polyvinylidene difluoride (PVDF) membrane (Immobilon-P, Merck Millipore, Billerica, MA, USA), blocked for 1 h at RT in 5% *w*/*v* non-fat milk in 1 X TBST (Tris-buffered saline, 0.1% Tween 20) and subsequently incubated overnight at 4 °C with the primary antibodies such as p-eIF2α, eIF2α (Cell Signaling Technology, Inc., Danvers, MA, USA) diluted in 5% *w*/*v* non-fat milk, 1 X TBST and 0.1% Tween 20. β-actin antibody (Cell Signaling Technology, Inc., Danvers, MA, USA) was used as a loading control. After probing with HRP-conjugated secondary antibody (Cell Signaling Technology, Inc., Danvers, MA, USA), diluted in 5% *w*/*v* non-fat milk, 1 X TBST and 0.1% Tween 20 for 1 h at RT, protein bands were visualized by enhanced chemiluminescence detection kit (Bio-Rad Laboratories, Hercules, CA, USA). After visualization, the X-ray films were scanned and the intensity of the protein bands was quantified using Gene Tools software (Syngene, Cambridge, UK).

### 4.4. Analysis of the Expression of ER Stress Markers

Total RNA was isolated from HTM cells using the PureLink RNA Mini Kit (Thermo Fisher Scientific Inc., Waltham, MA, USA) according to the manufacturer’s protocol. Isolated RNA was transcribed into cDNA to a final concentration of 100 ng using GoScript ™ Reverse Transcriptase (Promega Inc., Madison, WI, USA) according to the manufacturers protocol. Subsequently, we performed the TaqMan Gene Expression Assays to analyze the expression profile of genes related to ER stress conditions. For this purpose, we evaluated the level of the expression of three genes: *ATF4* (Hs00909569_g1), *DDIT3* (Hs01090850_m1) and *BAX* (Hs00180269_m1). As a reference gene, the *ACTB* (Hs99999903_m1) was used. For the qPCR analysis, the total reaction volume was 10 µL, that includes the following reagents: 1 uL cDNA, 1 µL primers, 2 µL 5x HOT FIREPol^®^ Probe qPCR Mix (Solis BioDyne, Tartu, Estonia) and 6 µL nuclease free water. Reaction conditions were used regarding to the manufacturers protocol: enzyme activation (15 min, 95 °C), denaturation (40 cycles, 10 s, 95 °C), annealing/extension (40 cycles, 60 s, 60 °C). Gene expression was performed using the Bio-Rad CFX96 (BioRad, Hercules, CA, USA) system.

### 4.5. Cytotoxicity Analysis

#### 4.5.1. XTT Assay

The cytotoxicity of the investigated PERK inhibitor LDN-0060609 was measured by the XTT colorimetric assay (Thermo Scientific, Waltham, MA, USA), that is used to assess cell viability as a function of redox potential. Actively respiring cells convert the water-soluble XTT to a water-soluble, orange colored formazan product. All experiments were performed in triplicate with similar results. HTM cells were seeded in 96-well poly-L-lysine-coated plates (5 × 10^3^/well) and cultured in 100 μL of complete TMCM growth medium for 24 h. After cell adhesion, cells were treated with 100 μL of complete cell culture medium containing LDN-0060609 investigated compound in a wide concentration range (0.75 μM, 3 μM, 6 μM, 12 μM, 25 μM, 50 μM, 75 μM, 100 μM, 50 mM) or 0.1% DMSO (Sigma-Aldrich Corp., St. Louis, MO, USA), which was used as a solvent for the investigated PERK inhibitor. Untreated cells cultured in a complete medium were used as a negative control, whereas cells incubated with 100% DMSO comprised a positive control. Moreover, to show the effect of the investigated PERK inhibitor after ER stress induction, HTM cells were seeded in 96-well poly-L-lysine-coated plates (5 × 10^3^/well) and cultured in 100 μL of complete TMCM growth medium for 24 h. After cell adhesion, cells were pretreated for 1 h with the 100 μL of complete cell culture medium containing PERK inhibitor LDN-0060609 at the concentrations of 3 μM and 25 μM, based on the data obtained in Western blot analysis, and then cells were treated with Th (500 nM), as an ER stress inducer. Cells were also treated with Th (500 nM) only. Untreated cells cultured in a complete medium were used as a negative control, whereas cells incubated with 100% DMSO comprised a positive control. All of the analyzed samples were incubated for 16, 24 and 48 h. Subsequently, 25 µL of XTT/PMS mixture was added to each well. After a 2 h incubation at 37 °C in a 5% CO_2_ incubator, absorbance was measured at a wavelength of 450 nm using Synergy HT (BioTek, Hong Kong, China) spectrophotometer.

#### 4.5.2. Lactate Dehydrogenase Assay

The release of LDH into the culture medium was assessed using a Pierce™ LDH Cytotoxicity Assay Kit (Thermo Scientific, Waltham, MA, USA). Extracellular LDH in the culture media is quantified by a coupled enzymatic reaction with LDH and catalyzes the conversion of lactate to pyruvate via NAD^+^ reduction to NADH. Subsequently, diaphorase uses NADH to reduce tetrazolium salt to a red formazan product measured spectrophotometrically. All experiments were performed in triplicate with similar results. HTM cells were seeded in a 96-well poly-L-lysine-coated plates (5 × 10^3^/well) and cultured in 100 μL of complete TMCM growth medium for 24 h. After cell adhesion, cells were treated with the investigated PERK inhibitory compound in a large concentration range (0.75 μM, 3 μM, 6 μM, 12 μM, 25 μM, 50 μM, 75 μM, 100 μM, 50 mM) or 0.1% DMSO (Sigma-Aldrich Corp., St. Louis, MO, USA). Furthermore, to examine the effect of the investigated PERK inhibitor after ER stress induction, HTM cells were seeded in 96-well poly-L-lysine-coated plates (5 × 10^3^/well) and cultured in 100 μL of complete TMCM growth medium for 24 h. After cell adhesion, cells were pretreated for 1 h with the 100 μL of complete cell culture medium containing PERK inhibitor LDN-0060609 at the concentrations of 3 μM and 25 μM and then cells were treated with Th (500 nM). Cells were also treated with Th (500 nM) Only. All of the analyzed samples were incubated for 16, 24 and 48 h. The maximum LDH activity was determined using the 10 X Lysis Buffer. For preparing PERK inhibitor concentrations and the maximum LDH activity control, cell culture medium with serum concentration reduced to 1% was used. After incubation, 50 μL of supernatant from each sample were transferred into new 96-well plates and subsequently 50 μL of the reaction mixture was added to each well. Following a 30 min incubation in the dark at room temperature (RT), the reaction was stopped via adding 50 μL of stop solution. Absorbance was measured at wavelengths of 490 nm and 680 nm (background) using the Synergy HT (BioTek, Hong Kong, China) spectrophotometer.

#### 4.5.3. Cell Survival Assay

HTM cells were seeded at 20,000 per 60 mm poly-L-lysine-coated dish and cultured in 4 mL of complete TMCM growth medium for 24 h. After cell adhesion, cells were treated with indicated doses (3–50 µM) of the PERK inhibitor LDN-0060609 or 0.1% DMSO (Sigma-Aldrich Corp., St. Louis, MO, USA), used as a solvent for the investigated PERK inhibitor, for 48 h. Untreated cells cultured in a complete medium were used as a negative control, whereas cells incubated with 10% DMSO comprised a positive control. Colony outgrowth was assessed by Giemsa (Sigma-Aldrich Corp., St. Louis, MO, USA) staining after 21 days. Growth media was changed every 3 days.

### 4.6. Genotoxicity Analysis

The genotoxicity of analyzed compound LDN-0060609 was evaluated using an alkaline version of the comet assay. A comet assay is a rapid and sensitive technique for quantifying and analyzing DNA damage on the level of individual cells. All experiments were performed in triplicate with similar results. Assays were prepared in poly-L-lysine-coated 6-well plates by adding 2 × 10^5^ cells in 2 mL of complete TMCM growth medium. After cell adhesion, cells were treated with the investigated PERK inhibitory compound in a wide concentration range (0.75 μM, 3 μM, 6 μM, 12 μM, 25 μM, 50 μM, 75 μM, 100 μM) or 0.1% DMSO (Sigma-Aldrich Corp., St. Louis, MO, USA), and incubated for 24 h and 48 h. Cells suspended in 5% DMSO (Sigma-Aldrich Corp., St. Louis, MO, USA), were used as a positive control, whereas cells suspended in 2 mL of complete TMCM growth medium were used as a negative control. To evaluate the effect of the PERK inhibitor LDN-0060609 in HTM cells with the induced ER stress conditions cells were seeded in poly-L-lysine-coated 6-well plates (2 × 10^5^ cells in 2 mL of complete TMCM growth medium). Next, cells were cultured in a complete TMCM growth medium for 24 h. After cells adhesion, they were pretreated for 1 h with the 2 mL of complete TMCM growth medium containing PERK inhibitor LDN-0060609 at the concentrations of 3 μM and 25 μM and then cells were treated with Th (50 nM). Cells were also treated only with Th (50 nM). Cells suspended in 5% DMSO (Sigma-Aldrich Corp., St. Louis, MO, USA), were used as a positive control, whereas cells suspended in 2 mL of complete TMCM growth medium were used as a negative control. All of the analyzed samples were incubated for 24 h and 48 h. Cell suspension in 0.37% LMP (low melting point) agarose (Sigma-Aldrich Corp., St. Louis, MO, USA) was placed on microscope slides previously coated with NMP (normal melting point) agarose (Sigma-Aldrich Corp., St. Louis, MO, USA). Preparations were incubated in lysis buffer at pH 10 (2.5-M NaCl, 10-mM Tris, 100-mM EDTA) and TritonX-100 (Sigma-Aldrich Corp., St. Louis, MO, USA) at a final concentration of 1% at 4 °C for 60 min. After 1 h of lysis, the preparations were incubated 20 min in development buffer (300-mM NaOH, 1-mM EDTA) at 4 °C, and then preparations were electrophoresed (32 mA, 17 V, 20 min) at 4 °C in electrophoretic buffer (30-mM NaOH, 1-mM EDTA). Subsequently, the preparations were rinsed three times with distilled water and then left to dry at RT. After staining with a fluorescent dye DAPI, preparations were analyzed under a fluorescent microscope. The value determining DNA damage in cells was the percentage of DNA in the comet tail.

### 4.7. Analysis of Morphological Changes

To evaluate the morphological changes of the HTM cells, with induced ER stress conditions, after their treatment with the investigated PERK inhibitor LDN-0060609 the phase-contrast microscopy has been used. HTM cells were seeded on the poly-L-lysine-coated T-75 culture vessels (1 × 10^6^ cells) and cultured in a complete TMCM growth medium for 24 h. After cell adhesion, cells were pretreated for 1 h with the PERK inhibitor LDN-0060609 in a large concentration range (3 μM, 6 μM, 12 μM, 25 μM, 50 μM) and then they were treated with Th (500 nM), as an ER stress inducer. Cells were also treated only with Th (500 nM). Untreated cells cultured in a complete TMCM growth medium were used as a negative control, whereas cells incubated with 100% DMSO comprised a positive control. Following 24 h and 48 h of incubation, the effect of the investigated PERK inhibitor on the morphological changes in HTM cells, with induced ER stress conditions, was assessed by an Leica inverted microscope at 40× magnification.

### 4.8. Apoptosis Analysis

#### 4.8.1. Caspase-3 Activity Assay

Caspase-3 activity was assessed using a colorimetric caspase-3 assay kit (Abcam, Cambridge, UK). Caspase-3 assay is based on the spectrophotometric detection of the chromophore p-nitroaniline (p-NA) after cleavage from the labeled substrate DEVD-p-NA. All experiments were repeated three times with similar results. HTM cells were seeded in 6-well poly-L-lysine-coated plates (5 × 10^5^/well) and cultured in a complete TMCM growth medium for 24 h. After cell adhesion, cells were treated with the PERK inhibitor LDN-0060609 in a large concentration range (3 μM, 6 μM, 12 μM, 25 μM, 50 μM) or 0.1% DMSO (Sigma-Aldrich Corp., St. Louis, MO, USA), and incubated for 24 h. Cells treated with staurosporine (Sigma-Aldrich Corp., St. Louis, MO, USA) at a 1 µM concentration for 16 h constituted a positive control, whereas cells incubated only in a complete medium for 24 h served as a negative control. To investigate the effect of the PERK inhibitor LDN-0060609 in HTM cells, with induced ER stress conditions, cells were seeded in 6-well poly-L-lysine-coated plates (5 × 10^5^/well) and cultured in a complete TMCM growth medium for 24 h. After cells adhesion, they were pretreated for 1 h with the complete TMCM cell culture medium containing PERK inhibitor LDN-0060609 at the concentrations of 3 μM and 25 μM and then cells were treated with Th (500 nM) for 24 h. Cells were also treated only with Th (500 nM) for 24 h. Cells treated with staurosporine (Sigma-Aldrich Corp., St. Louis, MO, USA) at a 1 µM concentration for 16 h constituted a positive control, whereas cells incubated for 24 h only in a complete medium served as a negative control. Following treatment and incubation with the investigated compounds, culture medium was removed, and cells were washed once with 1 X DPBS (Sigma-Aldrich Corp., St. Louis, MO, USA). Cells were detached after their exposure to 0.05% T/E solution (ScienCell Research Laboratories, San Diego, CA, USA). The resulting cell suspension was centrifuged for 5 min at 1000 rpm at RT. Subsequently, the supernatant was removed, cells were resuspended in a complete medium, counted, centrifuged for 5 min at 1000 rpm at RT and a pellet containing 1 × 10^6^ cells was re-suspended in 50 µL of cold Cell Lysis Buffer. After a 10 min incubation on ice, a suspension of cells was centrifuged at 10,000× *g* for 1 min. Supernatants were transferred to fresh 2 mL tubes and the protein concentration was measured by performing a standard Bradford assay. BSA was used as a protein standard. Cell lysate containing 100 μg protein was used for each assay. 2 X Reaction Buffer containing 10 mM DTT and subsequently 4 mM DEVD-pNA substrate (200 µM final concentration) were added to each sample. After a 2 h incubation at 37 °C, the p-NA absorbance was measured at a wavelength of 405 nm using the Synergy HT (BioTek, Hong Kong, China) spectrophotometer.

#### 4.8.2. Flow Cytometric Determination of Apoptosis by FITC Annexin V/Propidium Iodide Double Staining

Apoptotic cell death was measured by flow cytometry using the FITC Annexin V Apoptosis Detection Kit I purchased from BD Pharmingen™ (ApoAlert Annexin V, Clontech, CA, USA). The above-mentioned experimental method is based on FITC-conjugated Annexin V (Annexin V-FITC) that binds to phosphatidylserine at the cell surface of the apoptotic cells and the PI that constitutes a marker of cell membrane permeability. All experiments were performed in triplicate with similar results. HTM cells were double stained with Annexin V-FITC, as a marker of early apoptosis, and PI as a marker of cell membrane disintegration, necrosis and late apoptosis. HTM cells were seeded in 6-well poly-L-lysine-coated plates (5 × 10^5^/well) and cultured in a complete TMCM growth medium for 24 h. After cell adhesion, cells were treated with the investigated PERK inhibitory compound in a wide concentration range (3 μM, 6 μM, 12 μM, 25 μM, 50 μM) or 0.1% DMSO (Sigma-Aldrich Corp., St. Louis, MO, USA), and incubated for 24 h. Cells treated with staurosporine (Sigma-Aldrich Corp., St. Louis, MO, USA) at concentration of 1 µM for 16 h constituted a positive control, whereas cells incubated only in a complete medium for 24 h served as a negative control. Furthermore, to demonstrate the effect of the investigated PERK inhibitor LDN-0060609 in HTM cells with the induced ER stress conditions cells were seeded in 6-well poly-L-lysine-coated plates (5 × 10^5^/well) and cultured in a complete TMCM growth medium for 24 h. After cell adhesion, cells were pretreated for 1 h with the complete TMCM cell culture medium containing PERK inhibitor LDN-0060609 at a concentration of 3 μM and 25 μM and then cells were treated with Th (500 nM) and incubated for 24 h. Cells were also treated only with Th (500 nM) and incubated for 24 h. Cells treated with staurosporine (Sigma-Aldrich Corp., St. Louis, MO, USA) at concentration of 1 µM for 16 h constituted a positive control, whereas cells incubated only in a complete medium for 24 h served as a negative control. After treatment and incubation with the investigated compounds, culture medium was removed, and cells were washed once with 1 X DPBS (Sigma-Aldrich Corp., St. Louis, MO, USA). Cells were detached after their exposure to 0.05% T/E solution (ScienCell Research Laboratories, San Diego, CA, USA). The resulting cell suspension was centrifuged for 5 min at 1000 rpm at RT. Subsequently, the supernatant was removed and cells were washed twice with cold 1 X DPBS (Sigma-Aldrich Corp., St. Louis, MO, USA). The 10 X Annexin V Binding Buffer, composed of a 0.2 µm sterile filtered 0.1 M Hepes (pH 7.4), 1.4 M NaCl and 25 mM CaCl_2_ solution, was diluted with distilled water (1:10). Next, cells were re-suspended in 1 X Binding Buffer at a concentration of 1 × 10^6^ cells/mL, and then 100 µL of the cell suspension (1 × 10^5^ cells) was transferred to a 5 mL culture tube. 5 µL of FITC annexin V and 5 µL of PI were added to each analyzed sample. Cells were gently vortexed and incubated for 20 min at RT in the dark. Next, 200 µL of 1 X Binding Buffer was added to each sample. Cell suspensions were analyzed by flow cytometry using the Beckman Coulter CytoFLEX. The obtained data were analyzed using the Kaluza analysis 1.5 A software (Beckman Coulter, Brea, CA, USA).

### 4.9. Cell Cycle Analysis

The cell cycle analysis was carried out by flow cytometry using the PI staining. All experiments were performed in triplicate with similar results. HTM cells were seeded in 6-well poly-L-lysine-coated plates (5 × 10^5^/well) and cultured in a complete TMCM growth medium for 24 h. After cell adhesion, cells were treated with the PERK inhibitor LDN-0060609 in a wide concentration range (3 μM, 6 μM, 12 μM, 25 μM, 50 μM) or 0.1% DMSO (Sigma-Aldrich Corp., St. Louis, MO, USA), and incubated for 24 h. Cells treated with nocodazole (Sigma-Aldrich Corp., St. Louis, MO, USA) at a concentration of 1 µM for 16 h constituted a positive control, whereas cells cultured in complete medium for 24 h were used as a negative control. To investigate the effect of the PERK inhibitor LDN-0060609 in HTM cells with the induced ER stress conditions cells were seeded in 6-well poly-L-lysine-coated plates (5 × 10^5^/well) and cultured in a complete TMCM growth medium for 24 h. After cells adhesion, they were pretreated for 1 h with the complete TMCM cell culture medium containing PERK inhibitor LDN-0060609 at the concentrations of 3 μM and 25 μM and then cells were treated with Th (500 nM) and incubated for 24 h. Cells were also treated only with Th (500 nM) and incubated for 24 h. Cells treated with nocodazole (Sigma-Aldrich Corp., St. Louis, MO, USA) at a concentration of 1 µM for 16 h constituted a positive control, whereas cells cultured in complete medium for 24 h were used as a negative control. After treatment and incubation with the investigated compounds, cells were harvested and washed twice with cold 1 X DPBS (Sigma-Aldrich Corp., St. Louis, MO, USA). 1 × 10^6^ cells/mL were fixed with ice-cold 70% ethanol at −20 °C for 20 min. Subsequently, ethanol-suspended cells were centrifuged at 5000 rpm for 5 min. Pellets of cells were suspended in 250 µL of 1 X DPBS (Sigma-Aldrich Corp., St. Louis, MO, USA) and then cells were treated with RNase A, DNase & Protease-free (10 mg/mL) (Canvax Biotech, Córdoba, Spain) and incubated at 37 °C for 1 h before staining with PI solution (10 μg/mL) (Sigma-Aldrich Corp., St. Louis, MO, USA). After a 30 min incubation at 4 °C, samples were analyzed by flow cytometry using the Beckman Coulter CytoFLEX. The percentage of cells in each cell cycle phase, based on DNA content, was determined using Kaluza analysis 1.5 A software (Beckman Coulter, Brea, CA, USA). On the DNA content histograms the number of cells was plotted on the y-axis, whereas the DNA content, as measured by PI fluorescence, was depicted on the *x*-axis.

### 4.10. Statistical Analysis

Statistical analysis was performed using the Sigma Plot (Systat Software, Inc., San Jose, CA, USA). The normality test has been performed for each statistical analysis in all conducted studies using Shapiro-Wilk test. All statistical analyses, except comet assay test, were normally distributed, therefore the statistical analysis between two groups was performed using the Student’s *t*-test. In the comet assay analysis no normal distribution was obtained, therefore the statistical analysis between two groups was performed using the Mann-Whitney rank sum test. Each of the statistical analyses in individual experiments was based on the results of three independent tests. On the graphs, the differences were statistically significant as follows: * *p* < 0.05, ** *p* < 0.01, *** *p* < 0.001.

## 5. Conclusions

POAG, the most common type of glaucoma, constitutes a complex inherited eye disease, which is characterized by progressive RGCs death, optic nerve head excavation and subsequent visual field loss. However, according to the newest data, ER stress with the subsequent activation of the PERK-mediated UPR signaling pathway plays a key role in the POAG pathogenesis at the molecular level. Currently used treatment strategies against POAG are only symptomatic and limited mainly to the reduction of IOP. Thus, novel and effective treatment approaches for POAG are needed.

In this study, we have shown that small-molecule PERK inhibitor LDN-0060609 triggers significant inhibition of the eIF2α phosphorylation in HTM cells with induced ER stress conditions, that were used as an in vitro cellular model of POAG. In addition, treatment of HTM cells, with induced ER stress conditions, with the PERK inhibitor LDN-0060609 at the 25 μM concentration resulted in a significant decrease in the pro-apoptotic genes expression including *ATF4*, *DDIT3* and *Bax.* Moreover, the investigated PERK inhibitor LDN-0060609 demonstrated no cytotoxic and genotoxic effect toward HTM cells, as well as it did not induce apoptotic cell death or cell cycle arrest. The potency of the investigated PERK inhibitor LDN-0060609 has also been confirmed in the mentioned cellular model of POAG. Results obtained have shown that investigated LDN-0060609 compound at the concentration of 25 μM significantly undid the negative effect of Th-induced ER stress in HTM cell by increasing their viability and reducing DNA damage. In addition, treatment of HTM cells with Th-induced ER stress conditions with the increasing concentration of the PERK inhibitor LDN-0060609 undid the negative effect of Th-induced ER stress conditions by increasing cell proliferation and restoring normal cell morphology. In this analysis, the highest potency of PERK inhibitor LDN-0060609 was noticed at the 25 μM concentration. Furthermore, following incubation of HTM cells, with induced ER stress conditions, with the PERK inhibitor LDN-0060609 at a concentration of 25 μM, the percentage of apoptotic HTM cells was significantly decreased, as well as we have noticed a significant decrease in the percentage of cells in G2/M phase of the cell cycle as compared to only Th-treated HTM cells.

Thus, based on the latest literature data and results obtained from the present study, it can be concluded that targeting of the components of the PERK-dependent UPR signaling pathway via small-molecule PERK inhibitors, such as LDN-0060609, may contribute to the development of novel, innovative treatment strategies against POAG.

## Data Availability

The data that support the findings of this study are available from the corresponding author upon reasonable request.
